# Social Network Search for Solving Engineering Optimization Problems

**DOI:** 10.1155/2021/8548639

**Published:** 2021-09-30

**Authors:** Hadi Bayzidi, Siamak Talatahari, Meysam Saraee, Charles-Philippe Lamarche

**Affiliations:** ^1^Department of Civil Engineering, University of Tabriz, Tabriz, Iran; ^2^Engineering Faculty, Near East University, North Cyprus, Mersin 10, Turkey; ^3^Department of Civil and Building Engineering, Université de Sherbrooke, Sherbrooke, Canada

## Abstract

In this paper, a new metaheuristic optimization algorithm, called social network search (SNS), is employed for solving mixed continuous/discrete engineering optimization problems. The SNS algorithm mimics the social network user's efforts to gain more popularity by modeling the decision moods in expressing their opinions. Four decision moods, including imitation, conversation, disputation, and innovation, are real-world behaviors of users in social networks. These moods are used as optimization operators that model how users are affected and motivated to share their new views. The SNS algorithm was verified with 14 benchmark engineering optimization problems and one real application in the field of remote sensing. The performance of the proposed method is compared with various algorithms to show its effectiveness over other well-known optimizers in terms of computational cost and accuracy. In most cases, the optimal solutions achieved by the SNS are better than the best solution obtained by the existing methods.

## 1. Introduction

Optimization is a part of the nature of human works, in which almost all of the human decisions go through an optimal process [1]. Optimization is embedded in the essence of the many branches of science, for example, a system with minimal energy in physics, the maximum profit in business, survival of the best organism in biology, and designing an engineering system that satisfies a set of constraints [2, 3]. Almost all of the engineering problems contain several nonlinear and complex constraints depending on the design criteria and safety rules.

Over the last decades, various types of methods have been developed to solve constrained engineering problems. Two well-known groups of these methods are mathematical and metaheuristic methods. The idea of mathematical methods can be attributed to the development of the calculus of variations [4]. These methods employ the gradient of the objective function and constraints of the problem to find the optimal solution. The results of these methods are exact. However, these approaches search in a space near the starting point, which makes them sensitive to the initial starting point. In other words, just a correct starting point can lead to the global optima. In dealing with complex optimization problems, these methods are not suitable and frequently reach local solutions, and in some real applications, the gradient of the objective function and constraints is impossible to be calculated [5]. These drawbacks encourage researchers towards metaheuristic methods. Metaheuristic methods try to combine basic heuristic methods with randomization and rule-based theories, which are usually taken from natural phenomena such as evolution, swarm intelligence, and governing laws in different physics theories. Metaheuristic algorithms are approximate, but their results have high accuracy and are very close to the global optimum solution [6]. These methods are problem-independent, and the starting point does not determine the quality of the final solutions. Besides, these methods employ different operators to perform a global search in the space of the problem at an appropriate speed. These features have made them popular in recent decades. Also, these types of algorithms are among the most popular techniques that are employed for solving optimization problems in different fields, such as computer and electrical engineering [7], water, geotechnical and transport engineering [8], structure and infrastructures engineering [9], robotic [10], project and construction management [11], feature selection and data mining [12, 13], industrial and manufacturing [14], and medicine and biology [15].

**Figure 1:**
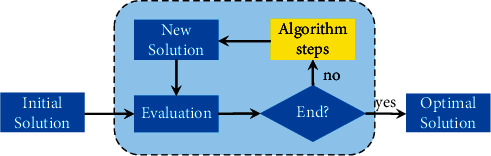
The general form of optimization algorithms.

Glover [16] introduced the term metaheuristic firstly. The word metaheuristic is a combination of two old Greek words: meta and heuristic. The word heuristic has its origin in the old Greek work heuriskein, which means the art of discovering new strategies (rules) to solve problems. The suffix meta also is a Greek word that means “upper-level methodology” [17]. Almost every metaheuristic algorithm follows the general process shown in Figure 1. Algorithm steps cause fundamental differences in the performance of algorithms when faced with different problems. In the other words, algorithm steps represent the unique operators of each algorithm in which new solutions are generated. The operators of each algorithm refer to the optimal process of a particular phenomenon that those algorithms have imitated.

According to the type of basic phenomena of each method, metaheuristic algorithms can be classified into four main categories: (1) evolutionary, (2) swarm intelligence, (3) physics-based, and (4) human-based algorithms. Evolutionary algorithms are motivated by natural evolution. Swarm intelligence algorithms model the natural behavior of animals in teamwork such as foraging and hunting. Physical phenomena and laws of science inspire physics-based algorithms, and finally, human-based algorithms mimic various optimal behaviors of humans in different conditions. Some of the most popular and novel algorithms are presented in Table 1.

Each of these algorithms can behave differently when dealing with different problems, so that one particular algorithm may not solve some problems. Therefore, it is necessary to create a new high-performance optimization algorithm that is able to solve more types of problems. Novel metaheuristic methods are developed to find the optimal solution for complex and large-scale problems in less time than previous ones, with higher accuracy. These aims are satisfied by developing more robust algorithms that have a better ability to search the space of problems to find a better solution. In addition, this property arises from the right balance between exploration and exploitation of the proposed algorithm. Exploitation means searching around the current best solutions, while exploration tries to explore the search space more efficiently, often by randomization [42].

In addition to inventing novel algorithms based on natural phenomena, developing new algorithms using hybridizing the operators of the current methods or modifying them is a hot topic in the field of metaheuristic algorithms. Firefly algorithm with chaos [43], hybrid particle swarm optimizer, ant colony strategy and harmony search scheme (HPSACO) [44], island-based cuckoo search with highly disruptive polynomial mutation (iCSPM) [45], quantum-behaved developed swarm optimizer (QDSO) [46], hybrid self-assembly with particle swarm optimization (SAPSO) [47], upgraded whale optimization algorithm (UWOA) [48], fuzzy controllers with slime mould algorithm (SMAF) [49], and hybrid invasive weed optimization-shuffled frog-leaping (SFLA-IWO) [50, 51] are some the newly developed hybrid or modified optimization algorithms.

Social network search (SNS) algorithm is a robust metaheuristic algorithm that was innovated as a novel method for solving optimization problems, and its results showed that it is capable of outperforming various methods in dealing with different optimization problems [42]. The SNS algorithm simulates human behavior as users of a social network. Social network users can influence the opinions of other users on the network by sharing their views, opinions, and thoughts. Each of the users can also share their thoughts on the network and affect other people's opinions. In other words, the SNS simulates particular moods where the views and opinions of users are influenced under their communications. This paper investigates the performance of the SNS algorithm using 14 constrained engineering optimization problems and a real application in the field of satellite image segmentation. The obtained results are compared with other optimizers in terms of best function value and number of function evaluations, and in most cases, the solutions of the SNS are better than the other methods.

The rest of this paper is organized as follows.  Section 2 describes the SNS algorithm and constraint-handling technique. The performance of the SNS algorithm in solving optimization problems is evaluated against other methods in Section 3. Finally, conclusions are given in Section 4.

## 2. Materials and Methods

This section presents the general framework of the SNS algorithm and the utilized constraint-handling technique for solving engineering optimization problems.

### 2.1. Social Network Search (SNS)

Human beings are social species, which always try to communicate with each other. Social networks are virtual tools that were created for this goal with the advent of technology. The proposed SNS algorithm simulates the interactive behavior among users in social networks to achieve more popularity. Social networks are platforms where users can interact virtually with other users. Interacting with other users of the network may affect their opinions. The process of interacting with and influencing other users of the network goes through an optimal process so that users are always trying to increase their level of popularity on the network.

**Figure 2:**
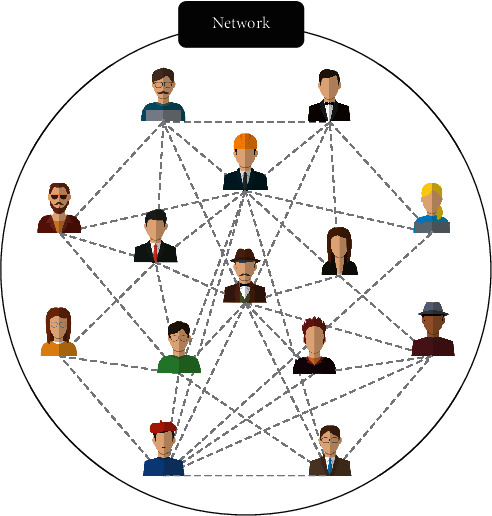
A general model for the social network.

The main property of social networks is that users can follow other persons, as shown in Figure 2. If a user shares a new post, that person's followers may be informed about the shared topic. This feature (fast propagation of views) has turned networks into a powerful tool for promoting information and ideas, which is due to having high connectivity of users in the social networks, as demonstrated in Figure 2.

In social networks, users' viewpoint can be affected by other views in different moods containing imitation, conversation, disputation, and innovation. One of these moods that look like real-world social behavior creates the new solution in the SNS algorithm. Description and mathematical modeling of these moods are as follows [42].

#### 2.1.1. Mood 1: Imitation

**Figure 3:**
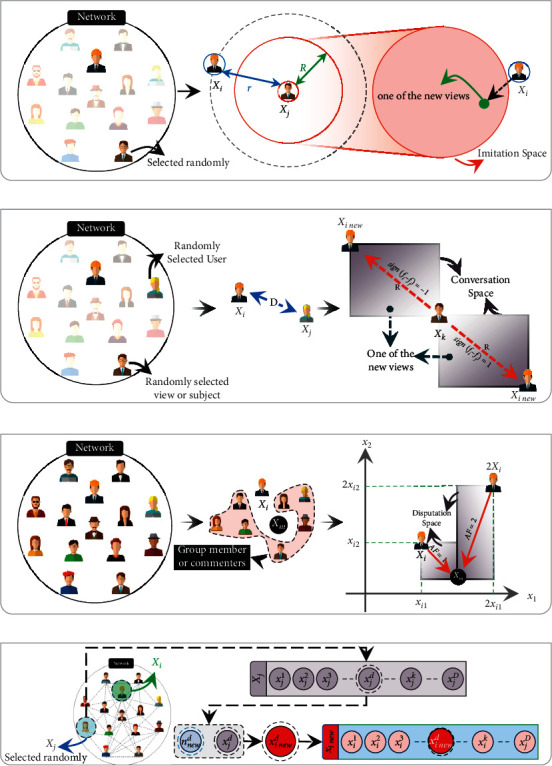
The process of different moods in the SNS algorithm. (a) Imitation. (b) Conversation. (c) Disputation. (d) Innovation.

Imitation means that the views of other users are attractive, and usually, users try to imitate each other in expressing their opinions as follows:

(1) $$\begin{array}{c} X_{i\text{new}}=\;X_{j}+\text{rand}\left(-1,1\right)\times R\text{,}\\ R=\text{rand }\left(0,1\right)\times r,\\ r=\;X_{j}-X_{i}, \end{array}$$

where *X*_*j*_ represents the vector of the *j*th user's view, which is selected randomly (*i* ≠ *j*), *X*_*i*_ is the view vector of the *i*th user, and rand(−1,1) and rand (0,1) are two random vectors in intervals [−1, 1] and [0, 1], respectively. In this mood, the new solution will be generated according to imitation space (Figure 3(a)), and this space is created using the radii of shock and popularity. The shock radius (*R*) reflects the amount of influence of the *j*th user, and its magnitude is considered as a multiple of *r*. The value of *r* shows the popularity radius of the *j*th user, which is calculated based on the difference in the opinions of the *i*th and *j*th users. Also, the final effect of the shock radius is reflected by multiplying its value to a random vector in the interval of [−1, 1], in which if the components of the random vector are positive, the shared view will be agreed with the *j*th opinion and vice versa. The process of the imitation mood is illustrated in Figure 3(a). As can be seen, using equation (1), the space of imitation will be formed, and then a point as a new view will be shared on the network.

#### 2.1.2. Mood 2: Conversation

In social networks, users can communicate with each other and benefit from their conversation about different issues according to

(2) $$\begin{array}{c} X_{i\text{new}}=\;X_{k}+R,\\ R=\text{ rand}\left(0,1\right)\times D,\\ D=\text{sign}\left(f_{i}-f_{j}\right)\times \left(X_{j}-X_{i}\right), \end{array}$$

where rand (0,1) is a random vector in the interval [0, 1], *X*_*j*_ and *X*_*k*_ are the vectors of two randomly selected positions somehow *i* ≠ *j* ≠ *k*, and  *f*_*i*_ and *f*_*j*_ are the objective functions of *X*_*i*_ and *X*_*j*_, respectively. This mood models a state in which users learn from each other and increase their information about events. In conversation, users find a sight about a specific issue through other views, and finally, due to the differences in opinions, they can draw a new vision of the issue under discussion. *X*_*k*_ demonstrates the vector of the issue, which is randomly chosen to speak about it; also, *R* is the effect of chat, which is based on the differences of opinion and represents the change in their beliefs about the issue. *D* is the difference between the views of users. In addition, sign(.) is the sign function and sign(*f*_*i*_ − *f*_*j*_) determines the moving direction of *X*_*k*_ by comparing *f*_*i*_ and *f*_*j*_. The process of this decision mood is shown in Figure 3(b). As can be seen, the user's view about the issue changes as a result of conversations with the *j*th user. The changed opinion is considered as a new view to share with others. Changing the user's view about the events is considered as the relocation of the events.

#### 2.1.3. Mood 3: Disputation

The disputation mood imagines a state where users explain their views about events to others and defend their opinion. In this situation, users see different views from other persons and may be influenced by the expressed reasons. The new affected view in disputation is as follows:

(3) $$\begin{array}{c} X_{i\text{new}}=X_{i}+\text{rand}\left(0,1\right)\times \left(M-AF\times X_{i}\right),\\ M=\frac{\sum_{t}^{N_{r}}X_{t}}{N_{r}},\\ \text{AF}=1+\text{round}\left(\text{rand}\right), \end{array}$$

where rand (0,1) is a random vector in the interval [0, 1] and *N*_*r*_ is a random integer between 1 and *N*_user_ (*N*_user_ is the population size or network size). *N*_*r*_ determines the number of users who participate in disputation, and participants are selected randomly. AF is the admission factor, which indicates the insistence from users on their opinion in discussions with other persons and is a random integer that can be either 1 or 2. round(.) is a function that rounds its input to the nearest integer number. The process of disputation mood is shown in Figure 3(c).

#### 2.1.4. Mood 4: Innovation

Sometimes a topic that users share on the networks comes from their new experiences and thoughts. In this mood, the new solution is developed by changing a randomly selected variable of *X*_*i*_ as follows:

(4) $$\begin{array}{c} X_{i\text{new}}=\left[x_{1},\;x_{2}\;,\;x_{3}\;,\;\ldots ,\;x_{inew}^{d}\;,\ldots \;,x_{D}\right],\\ x_{i\text{new}}^{d}=\;t\times x_{j}^{d}+\left(1-t\right)\times n_{\text{new}}^{d},\\ n_{\text{new}}^{d}=lb_{d}+{\text{rand}}_{1}\times \left(ub_{d}-lb_{d}\right),\\ t={\text{rand}}_{2}, \end{array}$$

where *d* is the *d*th variable that is selected randomly in the interval [1, *D*], *D* is the number of problem variables, and  rand_1_ and rand_2_ are two random numbers in the interval [0, 1]. Also, *ub*_*d*_ and *lb*_*d*_ are maximum and minimum values for the *d*th variable. *n*_new_^*d*^ represents the new idea about the selected dimension. *x*_*j*_^*d*^ is the current idea about the *d*th variable presented by another user (*j*th user which selected randomly and *i* ≠ *j*) that *i*th user wants to change it because of the new idea (*n*_new_^*d*^). Innovation models a state in which a person thinks about a specific issue, perhaps looks at that issue in a novel way, and is able to understand the nature of that problem more accurately or can find a completely different view about it. A particular subject may have distinct features, and each of them affects the understanding of the problem. As a result, by changing the idea about one of them (*x*_*i*_^*d*^), the general concept of the subject will change, and a novel view will be achieved. *x*_*i*new_^*d*^ is a new insight into the issue under consideration from the *d*th viewpoint and is replaced with the current view (*x*_*i*_^*d*^). The outline of the construction of the new view is shown in Figure 3(d).

In the SNS algorithm, only one of the predefined four models, so-called decision moods, will be selected and executed randomly for each user in each iteration of the algorithm. In other words, all of the moods described here are real-world behaviors of users in social networks, and it seems that the correct assumption is that only one of these moods occurs at a specific time (iteration) for each users. As a result, the chance of occurrence of these moods is considered to be small by using a random procedure with a uniform distribution.

**Figure 4:**
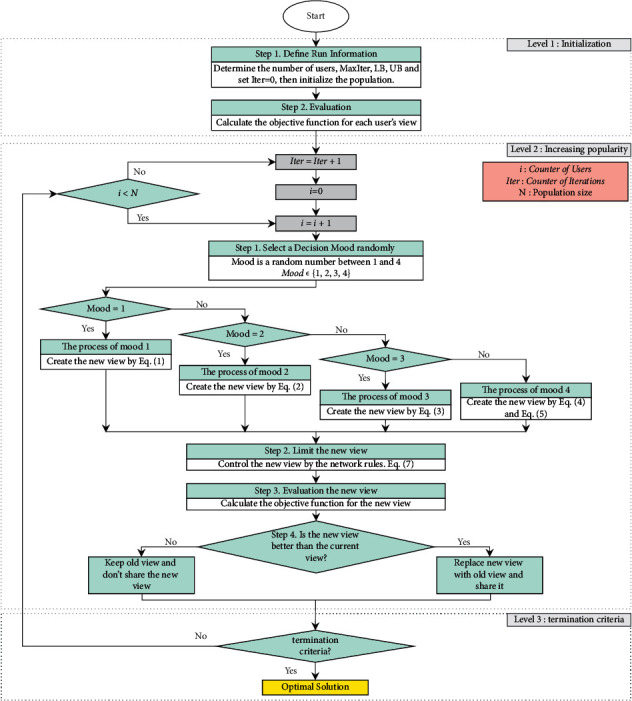
The flowchart of the SNS algorithm.

An important point is that the SNS algorithm has no specific parameter to be fine-tuned, and this feature is one of its superiority. In the third mood of the SNS algorithm, AF is defined as a random integer, and it can be considered a deterministic parameter whose value is generated randomly. To utilize the SNS algorithm, it just needs to determine the number of users (population size) and the maximum number of evaluations or iterations. The flowchart of the SNS algorithm is illustrated in Figure 4. Besides, the MATLAB code of the SNS algorithm for solving engineering optimization problems is available in [52].

### 2.2. Constraint-Handling Technique

Most engineering optimization problems aim to find optimal solutions under special conditions, which are usually based on resource limitations, design principles, and safety requirements. These special conditions are called constraints, and the main aim of constraint optimization is to find a feasible solution. A constrained optimization problems can be formulated as

(5) $$\begin{array}{c} \text{minimize}:\\ f\left(X\right),\;X=\left(x_{1},\;x_{2},\;\ldots ,\;x_{d}\right),\\ \text{subject to}:\\ g_{i}\left(X\right)\leq 0,\;\;i=1,\;2,\;\ldots ,\;n_{g},\\ h_{j}\left(X\right)=0,\;\;j=1,\;2,\;\ldots ,\;n_{h},\\ lb_{k}\leq \left(x_{k}\right)\leq ub_{k},\;\;k=1,\;2,\;\ldots ,\;d, \end{array}$$

where the function *f*(*X*) is an objective function, *X* is a vector of solution variables (and they can be continuous, discrete, or mixed), *g*_*i*_(*X*) and *h*_*j*_(*X*) are inequality and equality constraints, respectively, in which *n*_*g*_ is the number of inequality constraints and *n*_*h*_ is the number of equality constraints, *d* represents the number of variables, and *lb*_*k*_ and *ub*_*k*_ are the minimum and the maximum permissible values for the *k*th variable, respectively. The point worth mentioning is that a feasible solution satisfies all constraints. In contrast, infeasible solutions do not satisfy at least one constraint [53]. Also, in dealing with equality constraints, *h*_*j*_(*X*)=0 is replaced with an inequality |*h*_*j*_(*X*)| − *δ* ≤ 0, where *δ* is a positive tolerance value. Another approach for handling the equality constraints is to replace *h*_*j*_(*X*)=0 with two inequality constraints of the type *h*_*j*_(*X*) ≤ 0 and *h*_*j*_(*X*) ≥ 0. This strategy facilitates convergence to the optimum design [54]. Therefore, all of the constraints can be transformed into inequality constraints.

Metaheuristic algorithms cannot solve constraint optimization problems, directly. Therefore, it needs to equip them with an additional tool for handling constraints. A group of methods was developed for this proposal which is called constraint-handling techniques (CHTs). The CHTs enable the optimization methods to handle the objective function and constraints, simultaneously. CHTs are grouped into five categories: (1) penalty functions, (2) special representations and operators, (3) repair algorithms, (4) separation of objectives and constraints, and (5) hybrid methods [53]. The first method, penalty functions, is a simple and standard procedure for handling constraints. In the penalty function approach, a penalty term is added to the objective function, and then a constrained optimization problem is transformed into an unconstrained one. A penalty function can be formulated as follows:

(6) $$\begin{array}{c} F\left(X\right)=\;f\left(X\right)+P\left(X\right), \end{array}$$

where *F*(*X*) is the fitness function, which expresses the unconstrained state of the constrained problem, *f*(*X*) is the objective function, and *P*(*X*) is the penalty term that denotes the violation of constraints and is calculated as follows:

(7) $$\begin{array}{c} P\left(X\right)=\overset{n_{g}}{\underset{i=1}{\sum }}\alpha_{i}\times \max\left(0,\;g_{i}\left(X\right)\right)+\overset{n_{h}}{\underset{j=1}{\sum }}\beta_{j}\times \max\left(0,\left|\;h_{j}\left(X\right)\right|-\delta \right), \end{array}$$

where max(0,  *g*_*i*_(*X*)) and max(0, | *h*_*j*_(*X*)| − *δ*) represent the value of the violations of the solutions according to the *i*th inequality and *j*th equality constraints, respectively. Also, *α*_*i*_ and *β*_*j*_ are penalty factors for these constraints, respectively. The magnitude of penalty factors affects the quality of answers, and the suitable penalty factors are problem-dependent.

To solving constrained optimization problems, metaheuristic methods and CHT should be liked for recognizing feasible search space. Then, the optimizer should try to find the optimum or a near-optimum solution in the feasible region. Therefore, in each iteration of an algorithm, the fitness of the population is evaluated according to objective and constraint(s), and based on the calculated fitness function, the next generation of the population will be generated. In other words, the algorithm will identify the problem's search space using the fitness of the current population.

## 3. Results and Discussion

This section evaluates the performance of the SNS algorithm using 15 benchmark problems in various fields of engineering. One of these problems deals with the segmentation of satellite images in the field of remote sensing as a real application of metaheuristic algorithms in engineering. Each of these examples was run 30 times independently using the SNS algorithm, and the results are compared with different counterpart algorithms from the literature. In selecting the counterpart algorithm, an attempt has been made to use the results of newly developed methods.

### 3.1. Cantilever Beam

**Figure 5:**
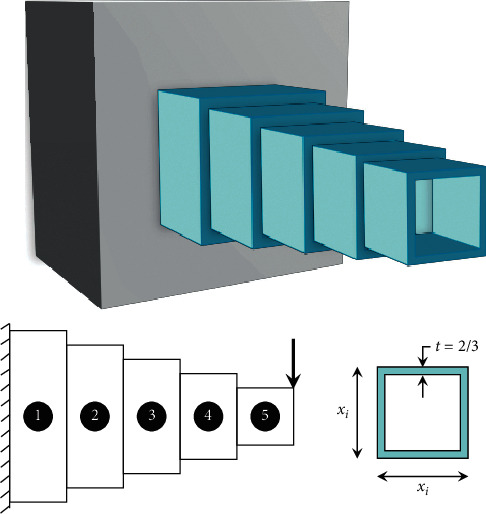
Schematic representation of cantilever beam.

This problem is a structural engineering design example that is related to the weight optimization of a cantilever beam with a square cross section [55]. The beam is rigidly supported at one end, and a vertical force acts on the free node of the cantilever, as shown in Figure 5. The beam consists of five hollow square blocks with constant thickness, whose heights (or widths) are the decision variables, and the thickness is held fixed (here 2/3). This problem can be expressed analytically as follows:

(8) $$\begin{array}{c} \text{minimize}:\\ f\left(X\right)=0.0624\left(x_{1}+x_{2}+x_{3}+x_{4}+x_{5}\right),\\ \text{subject to}:\\ g\left(X\right)=\frac{61}{x_{1}^{3}}+\frac{37}{x_{2}^{3}}+\frac{19}{x_{3}^{3}}+\frac{7}{x_{4}^{3}}+\frac{1}{x_{5}^{3}}-1\leq 0,\\ \text{variable range}:\\ 0.01\leq x_{i}\leq 100,\;\;i=1,\;\ldots ,\;5. \end{array}$$

The best solutions for solving this problem obtained by the SNS and various methods are listed in Table 2. It can be seen that the solution obtained by the SNS is better than that of the other methods. In addition, the SNS terminated after 12,000 evaluations. The statistical results of the SNS and other methods are listed in Table 3, and based on them, it can be seen that the SNS algorithm has obtained a more accurate answer in a smaller number of function evaluations (NFEs).

### 3.2. Optimal Design of I‐Shaped Beam

**Figure 6:**
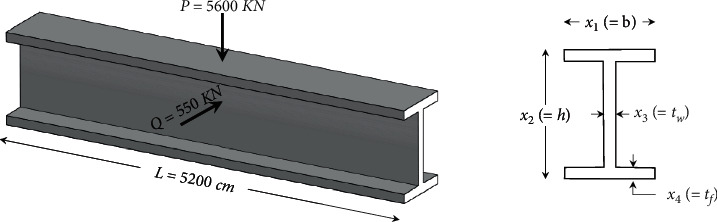
A 3D view of beam design problem.

The other typical engineering optimization problem is the I-beam design problem, which aims to minimize the vertical deflection of the beam shown in Figure 6. It simultaneously satisfies the cross-sectional area and stress constraints under given loads. The width of flange *b*(=*x*_1_), the height of section *h*(=*x*_2_), the thickness of the web  *t*_*w*_(=*x*_3_), and the thickness of the flange  *t*_*f*_(=*x*_4_)  are variables of this problem. The maximum vertical deflection of the beam is *f*(*x*) =PL^3^/48EI when the length of the beam (*L*) and modulus of elasticity (*E*) are 5200 cm and 523.104 kN/cm^2^, respectively. The objective function and constraints of this problem are formulated as follows:

(9) $$\begin{array}{c} \text{minimize}:\\ f\left(X\right)=\frac{5000}{x_{3}{\left(x_{2}-2x_{4}\right)}^{3}/12+\left(x_{1}x_{4}^{3}/6\right)+2bx_{4}{\left(x_{2}-x_{4}/2\right)}^{2}}\\ \text{subject to}:\\ g_{1}\left(X\right)=2x_{1}x_{3}+x_{3}\left(x_{2}-2x_{4}\right)\leq 300,\\ g_{2}\left(X\right)=\frac{18x_{2}\times 10^{4}}{x_{3}{\left(x_{2}-2x_{4}\right)}^{3}+2x_{1}x_{3}\left(4x_{4}^{2}+3x_{2}\left(x_{2}-2x_{4}\right)\right)}+\frac{15x_{1}\times 10^{3}}{\left(x_{2}-2x_{4}\right)x_{3}^{2}+2x_{3}x_{1}^{3}}\leq 56,\\ \text{variable range}:\\ 10\leq x_{1}\leq 50,\\ 10\leq x_{2}\leq 80,\\ 0.9\leq x_{3}\leq 5,\\ 0.9\leq x_{4}\leq 5. \end{array}$$

Many optimizers have solved this nonlinearly constrained problem, and Table 4 presents the best results of these methods. In addition, the statistical results for comparing the performance of the SNS methods are provided in Table 5. For this case study, the SNS needs 3600 function evaluations to reach these results, and it can be seen that the SNS performs superior compared to other methods.

### 3.3. Three-Bar Truss Design Problem

**Figure 7:**
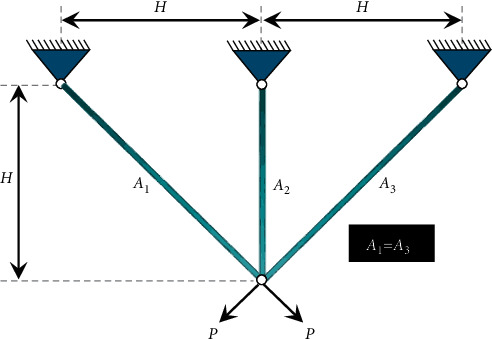
A schematic model of three-bar truss.

This case considers a 3-bar planar truss structure shown in Figure 7. The volume of a statically loaded 3-bar truss is to be minimized subject to stress (*σ*) constraints on each of the truss members. The objective is to evaluate the optimal cross-sectional areas, *A*_1_(=*x*_1_) and *A*_2_(=*x*_2_). The mathematical formulation is given below:

(10) $$\begin{array}{c} \text{minimize}:\\ f\left(X\right)=\left(2\sqrt{2}x_{1}+x_{2}\right)\times l,\\ \text{subject to}:\\ g_{1}\left(X\right)=\frac{\sqrt{2}x_{1}+x_{2}}{\sqrt{2}x_{1}^{2}+2x_{1}x_{2}}P-\sigma \leq 0,\\ g_{2}\left(X\right)=\frac{x_{2}}{\sqrt{2}x_{1}^{2}+2x_{1}x_{2}}P-\sigma \leq 0,\\ g_{3}\left(X\right)=\frac{1}{\sqrt{2}x_{2}+x_{1}}P-\sigma \leq 0,\\ l=100\text{ cm},\\ P=2\text{kN}/{\text{cm}}^{3},\\ \;\sigma =2\text{kN}/{\text{cm}}^{3},\\ \text{variable range}:\\ 0\leq x_{1},\\ \;x_{2}\leq 1. \end{array}$$

The best results of different methods are presented in Table 6. Also, Table 7 provides the statistical results of these algorithms. It can be seen that the best objective value of the SNS is equal or better than that of other methods. The required number of function evaluations (NFEs) for the SNS algorithm is 4800, which is much lower than that of other algorithms.

### 3.4. Tubular Column Design

**Figure 8:**
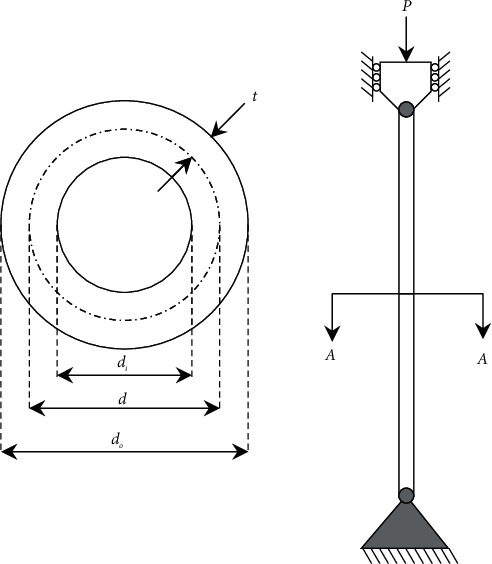
The 3D model of tubular column.

This problem is an example of designing a uniform column of the tubular section to carry a compressive load at minimum cost. This problem has two design variables, the mean diameter of the column *d*(=*x*_1_) and the thickness of tube *t*(=*x*_2_), which are shown in Figure 8. This column is made of a material with a yield stress of *σ*_*y*_=500 kgf/cm^2^ and a modulus of elasticity of *E*=0.85 × 10^6^6  kgf/cm^2^. The optimization model of this problem is given as follows:

(11) $$\begin{array}{c} \text{minimize}:\\ f\left(X\right)=9.8x_{1}x_{2}+2x_{1},\\ \text{subject to}:\\ g_{1}\left(X\right)=\frac{P}{\pi x_{1}x_{2}\sigma_{y}}-1\leq 0,\\ g_{2}\left(X\right)=\frac{8PL^{2}}{\pi^{3}Ex_{1}x_{2}\left(x_{1}^{2}+x_{2}^{2}\right)}-1\leq 0,\\ g_{3}\left(X\right)=\frac{2.0}{x_{1}}-1\leq 0,\\ g_{4}\left(X\right)=\frac{x_{1}}{14}-1\leq 0,\\ g_{5}\left(X\right)=\frac{0.2}{x_{2}}-1\leq 0,\\ g_{6}\left(X\right)=\frac{x_{2}}{8}-1\leq 0,\\ \text{variable range}:\\ 2\leq x_{1}\leq 14,\\ 0.2\leq x_{2}\leq 0.8. \end{array}$$

According to the constraints *g*_1_ and *g*_2_, the included stress in the column should be less than the buckling and yield stresses, respectively. Also, other constraints (*g*_3_, *g*_4_, *g*_5_, and *g*_6_) clamp the variables of the problem to the ranges of the variables. This problem was previously solved using various methods, and the best results of these methods and SNS are presented in Table 8. The SNS uses 1250 evaluations to solve this problem. In addition, the statistical results of some methods are reported in Table 9. According to these results, the SNS has found better results than other algorithms.

### 3.5. Speed Reducer Design

**Figure 9:**
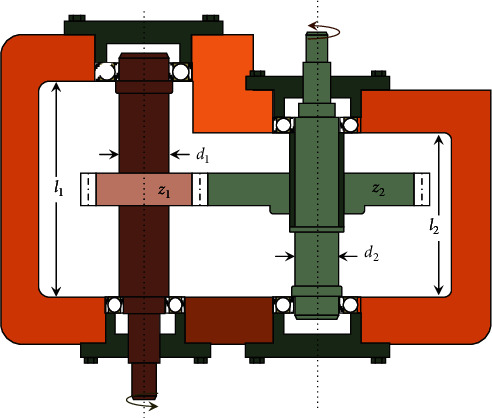
A schematic representation of speed reducer.

In mechanical systems, one of the essential parts of the gearbox is the speed reducer, and it can be employed for several applications [65]. In this optimization problem (see Figure 9), the weight of the speed reducer is to be minimized with subject to 11 constraints. This problem has seven variables, face width *b*(=*x*_1_), module of teeth *m*(=*x*_2_), the number of teeth in the pinion *z*(=*x*_3_), length of the first shaft between bearings *l*_1_(=*x*_4_), length of the second shaft between bearings *l*_2_(=*x*_5_), the diameter of first shafts *d*_1_(=*x*_6_), and the diameter of second shafts *d*_2_(=*x*_7_). The mathematical formulation of this problem is formulated as follows:

(12) $$\begin{array}{c} \text{minimize}:\\ f\left(X\right)=0.7854x_{1}x_{2}^{2}\left(3.3333x_{3}^{2}+14.9334x_{3}-43.0934\right)-1.508x_{1}\left(x_{6}^{2}+x_{7}^{2}\right)+7.4777\left(x_{6}^{3}+x_{7}^{3}\right)+0.7854\left(x_{4}x_{6}^{2}+x_{5}x_{7}^{2}\right),\\ \text{subject to}:\\ g_{1}\left(X\right)=\frac{27}{x_{1}x_{2}^{2}x_{3}}-1\leq 0,\\ g_{2}\left(X\right)=\frac{397.5}{x_{1}x_{2}^{2}x_{3}^{2}}-1\leq 0,\\ g_{3}\left(X\right)=\frac{1.93x_{4}^{3}}{x_{2}x_{6}^{4}x_{3}}-1\leq 0,\\ g_{4}\left(X\right)=\frac{1.93x_{5}^{3}}{x_{2}x_{7}^{4}x_{3}}-1\leq 0,\\ g_{5}\left(X\right)=\frac{\sqrt{{\left(745x_{4}/x_{2}x_{3}\right)}^{2}+16.9\times 10^{6}}}{110x_{6}^{3}}-1\leq 0,\\ g_{6}\left(X\right)=\frac{\sqrt{{\left(745x_{5}/x_{2}x_{3}\right)}^{2}+157.5\times 10^{6}}}{85x_{7}^{3}}-1\leq 0,\\ g_{7}\left(X\right)=\frac{x_{2}x_{3}}{40}-1\leq 0,\\ g_{8}\left(X\right)=\frac{5x_{2}}{x_{1}}-1\leq 0,\\ g_{9}\left(X\right)=\frac{x_{1}}{12x_{2}}-1\leq 0,\\ g_{10}\left(X\right)=\frac{1.5x_{6}+1.9}{x_{4}}-1\leq 0\\ g_{11}\left(X\right)=\frac{1.1x_{7}+1.9}{x_{5}}-1\leq 0,\\ \text{variable range}:\\ 2.6\leq x_{1}\leq 3.6,\\ 0.7\leq x_{2}\leq 0.8,\\ x_{3}\in \left\{17,\;18,\;19,\;\ldots ,\;28\right\},\\ 7.3\leq x_{4},\;\\ x_{5}\leq 8.3,\\ 2.9\leq x_{6}\leq 3.9,\\ 5\leq x_{7}\leq 5.5. \end{array}$$

This engineering problem has 11 constraints, seven nonlinear constraints and four linear inequality constraints, which are considered based on (1) bending stress of the gear teeth, (2) surface stress, (3) transverse deflections of the shafts, and (4) stresses in the shafts. The comparison of the best optimal solution with various optimization methods is given in Table 10. The SNS method requires 3750 evaluations to find its solution. The statistical results of SNS and ten optimization methods are compared in Table 11. Among the compared optimization algorithms, the SNS has the lowest number of function evaluations while its results are better than those of the other methods.

### 3.6. Piston Lever

**Figure 10:**
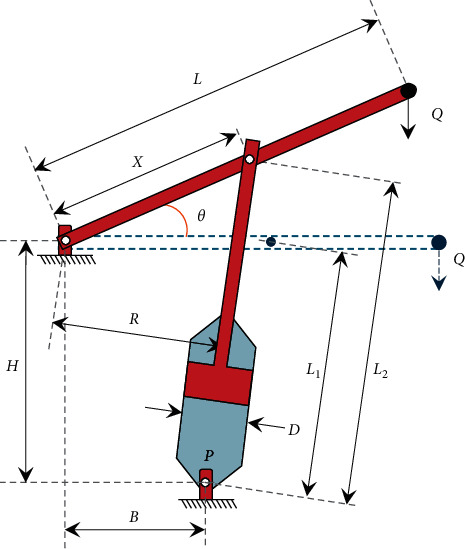
A model of piston lever problem.

The main objective of this problem is to locate the piston components, *H*(=*x*_1_), *B*(=*x*_2_), *D*(=*x*_3_), and *X*(=*x*_4_), by minimizing the oil volume when the lever of the piston is lifted up from ^0°^ to 45°, as shown in Figure 10. The formulation of this problem is given as follows:

(13) $$\begin{array}{c} \text{minimize}:\\ f\left(X\right)=\frac{1}{4}\pi x_{3}^{2}\left(L_{2}-L_{1}\right),\\ \text{subject to}:\\ g_{1}\left(X\right)=QL\text{cos}\theta -R\times F\leq 0,\\ g_{2}\left(X\right)=Q\left(L-x_{4}\right)-M_{\text{max}}\leq 0,\\ g_{3}\left(X\right)=1.2\left(L_{2}-L_{1}\right)-L_{1}\leq 0,\\ g_{4}\left(X\right)=\frac{x_{3}}{2}-x_{2}\leq 0,\\ \text{where}\\ R=\frac{\left|-x_{4}\left(x_{4}\text{sin}\theta +x_{1}\right)+x_{1}\left(x_{2}-x_{4}\text{cos}\theta \right)\right|}{\sqrt{{\left(x_{4}-x_{2}\right)}^{2}+x_{1}^{2}}},\\ F=\frac{\pi Px_{3}^{2}}{4},\\ L_{1}=\sqrt{{\left(x_{4}-x_{2}\right)}^{2}+x_{1}^{2}},\\ L_{2}=\sqrt{{\left(x_{4}\text{sin}\theta +x_{1}\right)}^{2}+{\left(x_{2}-x_{4}\text{cos}\theta \right)}^{2}},\\ \theta ={}^{45^{{}^{\circ}}},\\ Q=10,000\text{ lbs,}\\ L=240\text{ in,}\\ M_{\text{max}}=1.8\times 10^{6}\text{ lbs in,}\\ P=1500\text{ psi,}\\ \text{variable range}:\\ 0.05\leq x_{1},\;x_{2},\;x_{4}\leq 500,\\ 0.05\leq x_{3}\leq 120. \end{array}$$

These inequality constraints consider the force equilibrium, the maximum bending moment of the lever, minimum piston stroke, and geometrical conditions. The best solutions obtained by SNS and some of the other algorithms are presented in Table 12. In addition, the performance of the PSO [71], DE [71], GA [71], hybrid particle swarm optimization (HPSO) [71], HPSO with Q-learning [71], CS [18], ISA [63], CGO [37], MGA [40], AOS [39], and SNS is summarized in Table 13. The SNS algorithm obtains its results after 5000 evaluations, and its results are far better than those of other methods.

### 3.7. Corrugated Bulkhead Design

This problem aims to minimize the weight of a corrugated bulkhead in a chemical tanker [72], in which the design variables are the width (*x*_1_), depth (*x*_2_), length (*x*_3_), and plate thickness (*x*_4_). The mathematical model of this optimization problem is given as follows:

(14) $$\begin{array}{c} \text{minimize}:\\ f\left(X\right)=\frac{5.885x_{4}\left(x_{1}+x_{3}\right)}{x_{1}+\sqrt{\left|x_{3}^{2}-x_{2}^{2}\right|}},\\ \text{subject to}:\\ g_{1}\left(X\right)=-x_{4}x_{2}\left(0.4x_{1}+\frac{x_{3}}{6}\right)+8.94\left(x_{1}+\sqrt{\left|x_{3}^{2}-x_{2}^{2}\right|}\right)\leq 0,\\ g_{2}\left(X\right)=-x_{4}x_{2}^{2}\left(0.2x_{1}+\frac{x_{3}}{12}\right)+2.2{\left(8.94\left(x_{1}+\sqrt{\left|x_{3}^{2}-x_{2}^{2}\right|}\right)\right)}^{4/3}\leq 0,\\ g_{3}\left(X\right)=-x_{4}+0.0156x_{1}+0.15\leq 0,\\ g_{4}\left(X\right)=-x_{4}+0.0156x_{3}+0.15\leq 0,\\ g_{5}\left(X\right)=-x_{4}+1.05\leq 0,\\ g_{6}\left(X\right)=-x_{3}+x_{2}\leq 0,\\ \text{variable range}:\\ 0\leq x_{1},\;x_{2},\;x_{3}\leq 100,\\ 0\leq x_{4}\leq 5. \end{array}$$

Tables 14 and 15 compare the best and statistical results of SNS and other optimizers, respectively. According to these results, the SNS significantly improves the solution quality of other algorithms. In addition, the SNS method solves this problem after 3125 evaluations that is the lowest value among other methods.

### 3.8. Design of Pressure Vessel

**Figure 11:**
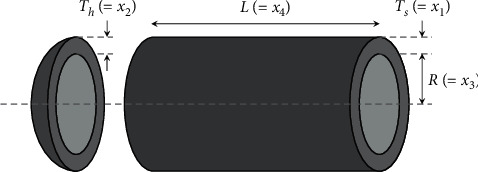
Schematic view of pressure vessel design.

A cylindrical vessel is capped at both ends by hemispherical heads, as shown in Figure 11. The objective is to minimize the total cost, including the cost of material, forming, and welding. This problem has four variables including the thickness of the shell  *T*_*s*_(=*x*_1_), the thickness of the head *T*_*h*_(=*x*_2_), the inner radius *R*=(*x*_3_), and the length of the cylindrical section of the vessel, not including the head *L*(=*x*_4_). In addition, *x*_1_ and *x*_2_ are integer multiples of 0.0625 in, while the other variables are continuous. The optimization problem can be expressed as follows:

(15) $$\begin{array}{c} \text{minimize}:\\ f\left(X\right)=0.6224x_{1}x_{3}x_{4}+1.7781x_{2}x_{3}^{2}+3.1661x_{1}^{2}x_{4}+19.84x_{1}^{2}x_{3},\\ \text{subject to}:\\ g_{1}\left(X\right)=-x_{1}+0.0193x_{3}\leq 0,\\ g_{2}\left(X\right)=-x_{2}+0.00954x_{3}\leq 0,\\ g_{3}\left(X\right)=-\pi x_{3}^{2}x_{4}-\frac{4}{3}\pi x_{3}^{3}+1,296,000\leq 0,\\ g_{4}\left(X\right)=x_{4}-240\leq 0,\\ \text{variable range}:\\ x_{1},\;x_{2}\in \left\{1\times 0.0625,\;2\times 0.0625,\;3\times 0.0625,\;\ldots ,\;1600\times 0.0625\right\},\\ 10\leq x_{3},\;\\ x_{4}\leq 200. \end{array}$$

This problem has been used to evaluate the performance of many algorithms. Tables 16 and 17 compare the best and statistical results of SNS and other algorithms, respectively. The SNS needs 6000 NFEs for solving this problem that is much lower than that of other algorithms.

### 3.9. Design of Tension/Compression Spring

**Figure 12:**
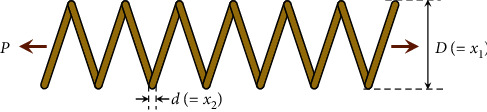
Tension/compression spring.

The tension/compression spring design problem is described in [81] for which the objective is to minimize the weight of a tension/compression spring, as shown in Figure 12. This problem is subject to constraints on minimum deflection, shear stress, surge frequency, limits on the outside diameter, and design variables. The design variables are the mean coil diameter *D* (=*x*_1_), the wire diameter *d* (=*x*_2_), and the number of active coils *N*(=*x*_3_). The problem can be stated as

(16) $$\begin{array}{c} \text{minimize}:\\ f\left(X\right)=\left(x_{3}+2\right)x_{2}x_{1}^{2},\\ \text{subject to}:\\ g_{1}\left(X\right)=1-\frac{x_{2}^{3}x_{3}}{71785x_{1}^{4}}\leq 0,\\ g_{2}\left(X\right)=\frac{4x_{2}^{2}-x_{1}x_{2}}{12566\left(x_{2}x_{1}^{3}-x_{1}^{4}\right)}+\frac{1}{5108x_{1}^{2}}-1\leq 0,\\ g_{3}\left(X\right)=1-\frac{140.45x_{1}}{x_{2}^{2}x_{3}}\leq 0,\\ g_{4}\left(X\right)=\frac{x_{1}+x_{2}}{1.5}-\leq 0,\\ \text{variable range}:\\ 0.05\leq x_{1}\leq 2,\\ 0.25\leq x_{2}\leq 1.3,\\ 2\leq x_{3}\leq 15. \end{array}$$

Table 18 compares the SNS with many optimization algorithms in terms of best optimization results, and Table 19 presents the statistical results of these algorithms. The SNS algorithm solves this problem in 9000 evaluations, and among the compared methods, just WOA [33] and MCEO [80] used a fewer number of evaluations, while their results are not as good as SNS.

### 3.10. Design of Welded Beam

**Figure 13:**
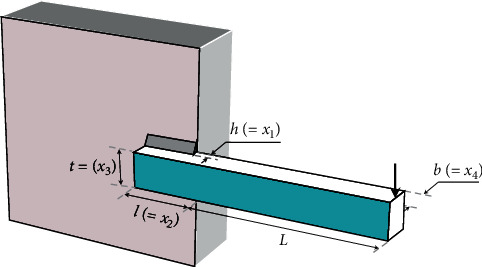
Schematic of the welded beam structure with indication of design variables.

This benchmark problem was introduced by Coello [77] and has been tackled by many researchers. As illustrated in Figure 13, the beam is under a vertical force. The goal of this problem is to find the minimum manufacturing cost of the welded beam. The problem is subject to seven constraints of stress, deflection, welding, and geometry. The variables are weld thickness *h*(=*x*_1_), height  *l*(=*x*_2_), length *t*(=*x*_3_), and bar thickness *b*(=*x*_4_), as shown in Figure 13. The objective function can be mathematically be stated as

(17) $$\begin{array}{c} \text{minimize}:\\ f\left(X\right)=1.10471x_{1}^{2}x_{2}+0.04811x_{3}x_{4}\left(14.0+x_{2}\right),\\ \text{subject to}:\\ g_{1}\left(X\right)=\tau \left(X\right)-\tau_{\text{max}}\leq 0,\\ g_{2}\left(X\right)=\sigma \left(X\right)-\sigma_{\text{max}}\leq 0,\\ g_{3}\left(X\right)=\delta \left(X\right)-\delta_{\text{max}}\leq 0,\\ g_{4}\left(X\right)=x_{1}-x_{4}\leq 0,\\ g_{5}\left(X\right)=P-P_{c}\left(X\right)\leq 0,\\ g_{6}\left(X\right)=0.125-x_{1}\leq 0,\\ g_{7}\left(X\right)=1.10471x_{1}^{2}+0.04811x_{3}x_{4}\left(14.0+x_{2}\right)-5.0\leq 0,\\ \tau \left(X\right)=\sqrt{{\left(\tau^{\prime }\right)}^{2}+2\tau^{\prime }\tau^{\prime \prime }\frac{x_{2}}{2R}+{\left(\tau^{\prime \prime }\right)}^{2},}\\ \tau^{\prime }=\frac{P}{\sqrt{2}x_{1}x_{2}},\\ \;\tau^{\prime \prime }=\frac{MR}{J},\\ M=P\left(L+\frac{x_{2}}{2}\right),\\ \;R=\sqrt{\frac{x_{2}^{2}}{4}+{\left(\frac{x_{1}+x_{3}}{2}\right)}^{2}},\\ \;J=2\left\{\sqrt{2}x_{1}x_{2}\left[\frac{x_{2}^{2}}{4}+{\left(\frac{x_{1}+x_{3}}{2}\right)}^{2}\right]\right\},\\ \sigma \left(\overset{⟶}{X}\right)=\frac{6PL}{x_{4}x_{3}^{2}},\;\\ \delta \left(\overset{⟶}{X}\right)=\frac{6PL^{3}}{Ex_{3}^{2}x_{4}},\\ \;P_{c}\left(\overset{⟶}{X}\right)=\frac{4.013E\sqrt{x_{3}^{2}x_{4}^{6}/36}}{L^{2}}\left(1-\frac{x_{3}}{2L}\sqrt{\frac{E}{4G}}\right),\\ \;P=6000\text{ lb},\\ \;L=14\text{ in},\;\\ \delta_{\text{max}}=0.25\text{ in},\;\\ E=30\times 10^{6}\text{ psi},\\ G=12\times 10^{6}\text{ psi},\\ \;\tau_{\text{max}}=13,600\text{ psi},\;\\ \sigma_{\text{max}}=30,000\text{ psi}.\\ \text{variable range}:\\ 0.1\leq x_{1},\\ x_{4}\leq 2,\\ 0.1\leq x_{2},\\ x_{3}\leq 10. \end{array}$$

Tables 20 and 21 compare the best and statistical results of various optimizer in dealing with welded beam design problem. The SNS algorithm needs 9000 evaluations, which is the lowest NFE among other algorithms, while its results are better. In addition, the SNS algorithm has the lowest standard deviation that shows its robustness in solving this problem.

### 3.11. Design of Gear Train

**Figure 14:**
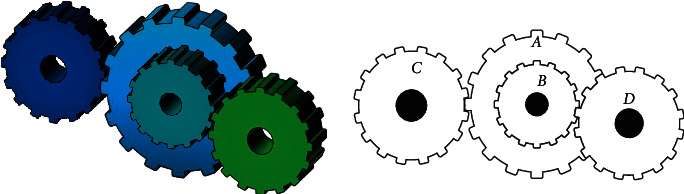
The 3D model of gear train.

The gear train design problem is an unconstrained discrete design problem in mechanical engineering and was introduced by Sandgren [85]. The purpose of this benchmark task is to minimize the gear ratio defined as the ratio of the angular velocity of the output shaft to the angular velocity of the input shaft. The number of teeth of gears *n*_*A*_(=*x*_1_), *n*_*B*_(=*x*_2_), *n*_*C*_(=*x*_3_), and *n*_*D*_(=*x*_4_) are considered as the design variables, and Figure 14 illustrates the 3D model of this problem. The mathematical formulation is provided as follows:

(18) $$\begin{array}{c} \text{minimize}:\\ f\left(X\right)={\left(\frac{1}{6.931}-\frac{x_{3}x_{2}}{x_{1}x_{4}}\right)}^{2},\\ \text{variable range}:\\ x_{1},\;x_{2},\;x_{3},\;x_{4}\in \left\{12,\;13,\;14,\;\ldots ,\;60\right\}. \end{array}$$

The best results of 19 algorithms include the SNS are presented in Table 22. It can be seen that all the algorithms find the optimum solution, except the PSO [62] and BBO [62]. In addition, the statistical results of 14 algorithms are compared in Table 23. The proposed method outperformed most of the other algorithms in terms of the mean, worst, SD, and NFEs.

### 3.12. A Reinforced Concrete Beam Design

**Figure 15:**
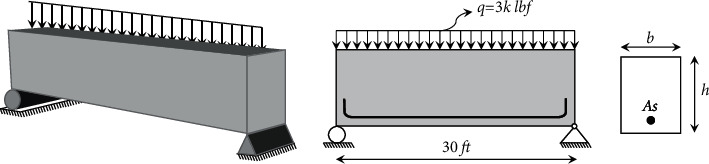
A schematic view of reinforced concrete beam.

Amir and Hasegawa [91] presented a simplified optimization problem of designing a reinforced concrete beam, as shown in Figure 15. The beam is assumed to be simply supported with a span of 30 ft and subjected to a live load of 2000 lbf and a dead load of 1000 lbf, including the weight of the beam. The concrete compressive strength (*σ*_*c*_) is 5 ksi, and the yield stress of the reinforcing steel (*σ*_*y*_) is 50 ksi. The cost of concrete is $0.02/in2/linear ft, and the cost of steel is $1.0/in2/linear ft. To minimize the total cost of the structure, the area of the reinforcement *A*_*s*_(=*x*_1_), the width of the beam *b*(=*x*_2_), and the depth of the beam *h*(=*x*_3_) have to be determined. The structure should be proportioned to have a required strength based upon the ACI building code 318-77 as follows:

(19) $$\begin{array}{c} M_{u}=0.9A_{s}\sigma_{y}\left(0.8h\right)\left(1.0-0.59\frac{A_{s}\sigma_{y}}{0.8bh\sigma_{c}}\right)\geq 1.4M_{d}+1.7M_{l}, \end{array}$$

where *M*_*u*_, *M*_*d*_, and *M*_*l*_ are the flexural strength, dead load, and live load moments of the beam, respectively. In this case, *M*_*d*_=1350 in kip  and  *M*_*l*_=2700 in kip. The depth to width ratio of the beam is restricted to be less than or equal to 4. The optimization problem can be expressed as

(20) $$\begin{array}{c} \text{minimize}:\\ f\left(X\right)=2.9x_{1}+0.6x_{2}x_{3},\\ \text{subject to}:\\ g_{1}\left(X\right)=\frac{x_{2}}{x_{3}}-4\leq 0,\\ g_{2}\left(X\right)=180+7.375\frac{x_{1}^{2}}{x_{3}}-x_{1}x_{2}\leq 0,\\ \text{variable range}:\\ x_{1}\in \left\{6,\;6.16,\;6.32,\;6.6,\;7,\;7.11,\;7.2,\;7.8,\;7.9,\;8,\;8.4\right\},\\ x_{2}\in \left\{28,\;29,\;30,\;\ldots ,\;40\right\},\\ 5\leq x_{3}\leq 10. \end{array}$$

It is clear that the variables *x*_1_ and *x*_2_ are discrete, while *x*_3_ is continuous. The SNS method requires1000 evaluations to reach the optimum solution.  Table 24 presents the results of optimum designs obtained by the SNS and other methods for this problem. In addition, the statistical results of FA [54], CS [18], AOS [39], and SNS are compared in Table 25. Obviously, the performance of the SNS method is better than other algorithms.

### 3.13. Car Side Impact Design

**Figure 16:**
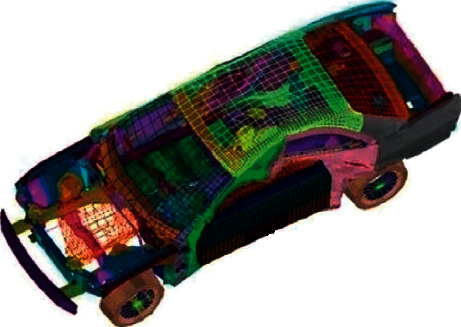
A model of car side impact problem.

On the foundation of the European Enhanced Vehicle-Safety Committee (EEVC) procedures, a car is exposed to a side impact, and the aim of this benchmark problem is minimizing the weight of the door using nine influence parameters including thicknesses of B-pillar inner (=*x*_1_), B-pillar reinforcement (=*x*_2_), floor side inner (=*x*_3_), cross members (=*x*_4_), door beam (=*x*_5_), door beltline reinforcement (=*x*_6_), roof rail (=*x*_7_), materials of B-pillar inner (=*x*_8_), floor side inner (=*x*_9_), barrier height (=*x*_10_), and hitting position (=*x*_11_). Youn et al. [93] simplified this optimization problem's analytical formulation and sped up computations using the global response surface methodology to approximate the structural weight and response to the impact.  Figure 16 shows a model for the car side impact problem. According to the simplified models, the optimization problem is formulated as follows:

(21) $$\begin{array}{c} \text{minimize}:\\ f\left(X\right)=1.98+4.90x_{1}+6.67x_{2}+6.98x_{3}+4.01x_{4}+1.78x_{5}+2.73x_{7},\\ \text{subject to}:\\ g_{1}\left(X\right)=1.16-0.3717x_{2}x_{4}-0.00931x_{2}x_{10}-0.484x_{3}x_{9}+0.01343x_{6}x_{10}-1\leq 0,\\ g_{2}\left(X\right)=46.36-9.9x_{2}-12.9x_{1}x_{2}+0.1107x_{3}x_{10}-32\leq 0,\\ g_{3}\left(X\right)=33.86+2.95x_{3}+0.1792x_{3}-5.057x_{1}x_{2}-11.0x_{2}x_{8}-0.0215x_{5}x_{10}-9.98x_{7}x_{8}+22.0x_{8}x_{9}-32\leq 0,\\ g_{4}\left(X\right)=28.98+3.818x_{3}-4.2x_{1}x_{2}+0.0207x_{5}x_{10}+6.63x_{6}x_{9}-7.7x_{7}x_{8}+0.32x_{9}x_{10}-32\leq 0,\\ g_{5}\left(X\right)=0.261-0.0159x_{1}x_{2}-0.188x_{1}x_{8}-0.019x_{2}x_{7}+0.0144x_{3}x_{5}+0.0008757x_{5}x_{10}+0.08045x_{6}x_{9}+0.00139x_{8}x_{11}+0.00001575x_{10}x_{11}-0.32\leq 0,\\ g_{6}\left(X\right)=0.214+0.00817x_{5}-0.131x_{1}x_{8}-0.0704x_{1}x_{9}+0.03099x_{2}x_{6}-0.018x_{2}x_{7}+0.0208x_{3}x_{8}+0.121x_{3}x_{9}-0.00364x_{5}x_{6}+0.0007715x_{5}x_{10}-0.0005354x_{6}x_{10}+0.00121x_{8}x_{11}+0.00184x_{9}x_{10}-0.02x_{2}^{2}-0.32\leq 0,\\ g_{7}\left(X\right)=0.74-0.61x_{2}-0.163x_{3}x_{8}+0.001232x_{3}x_{10}-0.166x_{7}x_{9}+0.227x_{2}^{2}-0.32\leq 0,\\ g_{8}\left(X\right)=4.72-0.5x_{4}-0.19x_{2}x_{3}-0.0122x_{4}x_{10}+0.009325x_{6}x_{10}+0.000191x_{11}^{2}-4\leq 0,\\ g_{9}\left(X\right)=10.58-0.674x_{1}x_{2}-1.95x_{2}x_{8}+0.02054x_{3}x_{10}-0.0198x_{4}x_{10}+0.028x_{6}x_{10}-9.9\leq 0,\\ g_{10}\left(X\right)=16.45-0.489x_{3}x_{7}-0.843x_{5}x_{6}+0.0432x_{9}x_{10}-0.0556x_{9}x_{11}-0.000786x_{11}^{2}-15.7\leq 0,\\ \text{variable range}:\\ 0.5\leq x_{1},\;x_{2},\;x_{3},\;x_{4},\;x_{5},\;x_{6},\;x_{7}\leq 1.5,\\ x_{8},x_{9}\in \left\{0.192,\;0.345\right\},,\\ -30\leq x_{10},\\ x_{11}\leq +30. \end{array}$$

The design of car side impact is also used as a benchmark problem to evaluate the performance of various methods. The best results of the SNS and these algorithms are presented in Table 26. It should be noted that the results of other algorithms evaluated by Gandomi et al. [18, 54, 66] have different variables ranges, but in this paper, the variable ranges of [93] are utilized.  Table 27 summarizes the statistical results obtained by the different optimization algorithms for the car side impact design problem. In this case, the SNS achieves its results with 20,000 NFEs. The CS method has better performance than all of the methods, according to the results presented in [18]. In addition, the best result of the SNS is better than those of other algorithms.

### 3.14. Cantilever Stepped Beam

**Figure 17:**
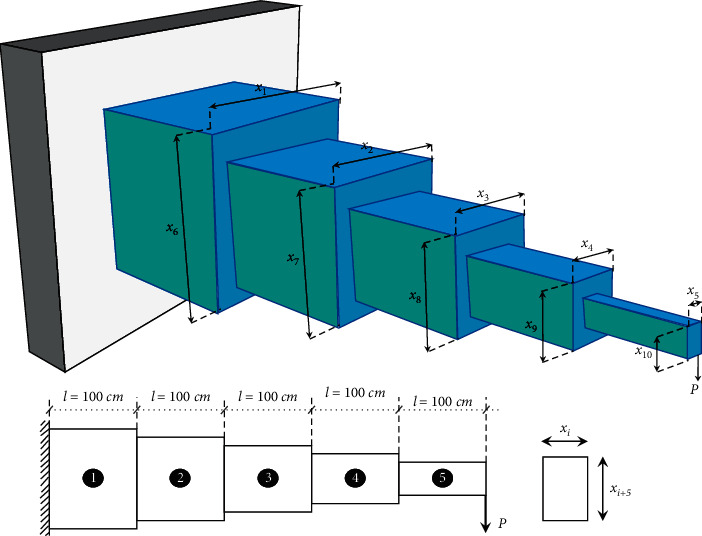
A 3D model of stepped cantilever beam.

This problem is a good benchmark to verify the capability of the optimization methods for solving continuous, discrete, and mixed variable structural design problems. This problem aims to minimize the volume of the beam. The width of segments (*x*_1_, *x*_2_, *x*_3_, *x*_4_, *x*_5_) and height of them (*x*_6_, *x*_7_, *x*_8_, *x*_9_, *x*_10_) are chosen to be the design variables. These ten variables are illustrated in Figure 17. Except for bending stress constraints, a specified aspect ratio is imposed such that the ratio of height to width in the segments of the beam is limited to be less than 20. The problem is formulated as follows:

(22) $$\begin{array}{c} \text{minimize}:\\ f\left(X\right)=D\left(x_{1}x_{6}l_{1}+x_{2}x_{7}l_{2}+x_{3}x_{8}l_{3}+x_{4}x_{9}l_{4}+x_{5}x_{10}l_{5}\right),\\ \text{subject to}:\\ g_{1}\left(X\right)=\frac{6Pl_{5}}{x_{5}x_{10}^{2}}-\sigma_{d}\leq 0,\\ g_{2}\left(X\right)=\frac{6P\left(l_{5}+l_{4}\right)}{x_{4}x_{9}^{2}}-\sigma_{d}\leq 0,\\ g_{3}\left(X\right)=\frac{6P\left(l_{5}+l_{4}+l_{3}\right)}{x_{3}x_{8}^{2}}-\sigma_{d}\leq 0,\\ g_{4}\left(X\right)=\frac{6P\left(l_{5}+l_{4}+l_{3}+l_{2}\right)}{x_{2}x_{7}^{2}}-\sigma_{d}\leq 0,\\ g_{5}\left(X\right)=\frac{6P\left(l_{5}+l_{4}+l_{3}+l_{2}+l_{1}\right)}{x_{1}x_{6}^{2}}-\sigma_{d}\leq 0,\\ g_{6}\left(X\right)=\frac{Pl^{3}}{3E}\left(\frac{1}{I_{5}}+\frac{1}{I_{4}}+\frac{1}{I_{3}}+\frac{1}{I_{2}}+\frac{1}{I_{1}}\right)-\Delta_{max}\leq 0,\\ g_{7}\left(X\right)=\frac{x_{10}}{x_{5}}-20\leq 0,\\ g_{8}\left(X\right)=\frac{x_{9}}{x_{4}}-20\leq 0,\\ g_{9}\left(X\right)=\frac{x_{8}}{x_{3}}-20\leq 0,\\ g_{10}\left(X\right)=\frac{x_{7}}{x_{2}}-20\leq 0,\\ g_{11}\left(X\right)=\frac{x_{6}}{x_{1}}-20\leq 0,\\ P=50000N,\\ \;\sigma_{d}=14,000N/{\text{cm}}^{2},\;\\ E=2\times 10^{7}N/{\text{cm}}^{2},\;\\ \Delta_{\text{max}}=2.7\text{ cm},\;\\ D=1.0,\\ \text{variable range}:\\ x_{1}\in \left\{1,\;2,\;3,\;4,\;5\right\},\\ x_{2},x_{3}\in \left\{2.4,\;2.6,\;2.8,\;3.1\right\},\\ 1\leq x_{4},\;\\ x_{5}\leq 5,\\ x_{6}\in \left\{30,\;31,\;32,\;\ldots ,\;65\right\},\\ x_{7},x_{8}\in \left\{45,\;50,\;55,\;60,\;65\right\},\\ 30\leq x_{9},\;\\ x_{10}\leq 65. \end{array}$$

The first five constraints are related to the bending stresses in each beam segment that must be lower than the allowable limit (*σ*_*d*_). Also, the deflection of the cantilever beam tip must be smaller than the limit deflection (Δ_max_). The aspect ratio between the height and width of the cross sections must be less than 20 and is applied by the last five constraints. Six of the variables (*x*_1_, *x*_2_, *x*_3_, *x*_6_, *x*_7_, *x*_8_) are discrete, and the rest of them (*x*_4_, *x*_5_, *x*_9_, *x*_10_) are continuous.

The best and statistical results of the FA [54], thermal exchange optimization (TEO) [82], PSO [82], and SNS are presented in Tables 28 and 29, respectively. To solve this problem, the SNS needs 20,000 evaluations, which is lower than the NFEs of other compared methods, and at the same time, it outperforms all of them in terms of best, mean, worst, and SD.

### 3.15. Real Application of SNS in Remote Sensing (Segmentation of Satellite Images)

Image segmentation is an important topic in the field of remote sensing due to the increasing volume of collected images from satellites, airplanes, and other platforms [95]. Image segmentation aims to partition an image into several homogenous sections such that the combination of no two adjacent sections is homogenous. Segmentation is a difficult task due to poor resolution, unfavorable environmental conditions, ambiguous regions, and the presence of pixels with a weak local correlation in satellite images [96, 97]. Metaheuristic methods are proper tools that can deal with the difficulty of discovering the homogeneity measure in the images [98]. In this section, the SNS algorithm is employed for segmenting color satellite images, and then its results have been compared with different optimization algorithms.

Thresholding techniques are the most common methods that are used as the objective function in image segmentation. In this study, Kapur's entropy method is employed for this purpose, and its mathematical formulation is as follows:

(23) $$\begin{array}{c} H_{0}=-\overset{t_{1}-1}{\underset{i=0}{\sum }}\left(\frac{p_{i}}{\omega_{0}}\right)\log_{2}\left(\frac{p_{i}}{\omega_{0}}\right);\\ H_{1}=-\overset{t_{2}-1}{\underset{i=t_{1}}{\sum }}\left(\frac{p_{i}}{\omega_{1}}\right)\log_{2}\left(\frac{p_{i}}{\omega_{1}}\right);\\ H_{j}=-\overset{t_{j+1}-1}{\underset{i=t_{j}}{\sum }}\left(\frac{p_{i}}{\omega_{j}}\right)\log_{2}\left(\frac{p_{i}}{\omega_{j}}\right);\\ H_{m}=-\overset{N-1}{\underset{i=t_{m}}{\sum }}\left(\frac{p_{i}}{\omega_{m}}\right)\log_{2}\left(\frac{p_{i}}{\omega_{m}}\right), \end{array}$$

where

(24) $$\begin{array}{c} \omega_{0}=\sum_{i=0}^{t_{1}-1}p_{i};\\ \omega_{1}=\sum_{i=t_{1}}^{t_{2}-1}p_{i};\\ \omega_{j}=\sum_{i=t_{j}}^{t_{j+1}-1}p_{i};\\ \omega_{m}=\sum_{i=t_{m}}^{N-1}p_{i}, \end{array}$$

where *H*_0_,  *H*_1_,   …,  *H*_*m*_ are the entropy values of *m* + 1 various sections or classes, *p*_*i*_ is the probability of the pixel intensity, and *N* is the total number of distinct intensity levels.

**Figure 18:**
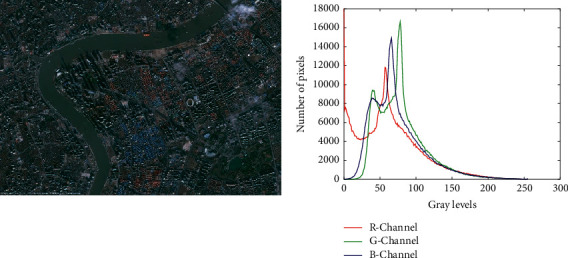
The utilized satellite image and its histograms (https://www.satpalda.com/gallery).

The utilized image and its histogram patterns are shown in Figure 18. This satellite image is taken from *Pléiades Satellite Imagery* to carry out the experimental study for segmentation. It can be seen that the histogram of the satellite image has a multimodal pattern, and it is very difficult to segment such an image that possesses immense information content.

**Figure 19:**
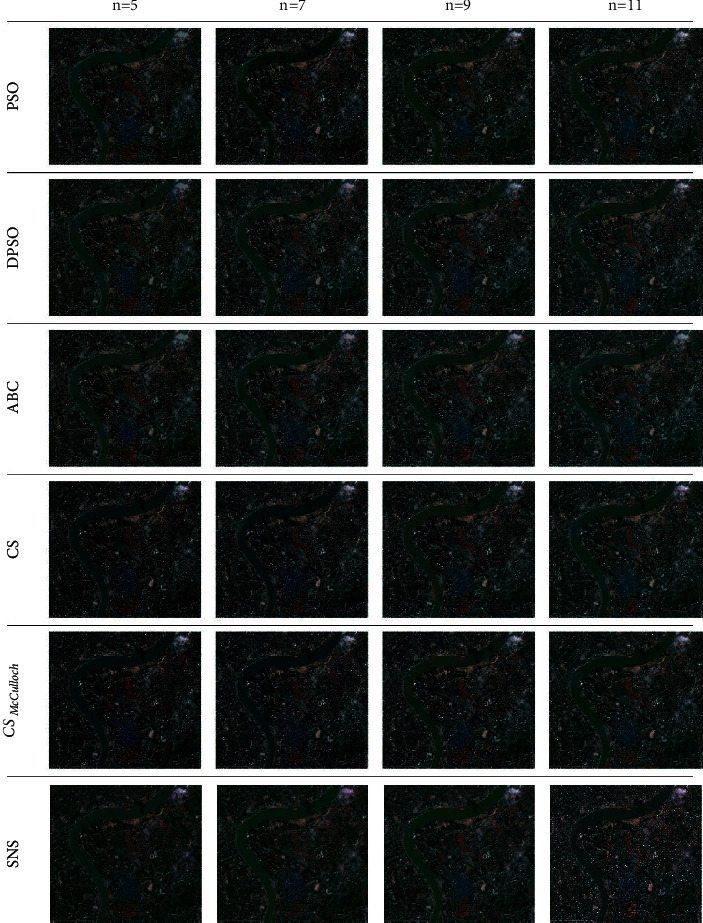
Segmentation results of test images using metaheuristic algorithms for four different threshold levels.

The experiment was carried out 10 times to choose the best of each algorithm.  Figure 19 gives the segmented images for four different levels of thresholding (*n*) and compares the results of the SNS algorithm with PSO, Darwinian PSO (DPSO), ABS, CS, and CS algorithm with McCulloch's method (CS_McCulloch_). Also, Table 30 presents the comparison of threshold values between the SNS and other methods.

Various criteria can be used for comparing the performance of metaheuristic algorithms in satellite image segmentation. Peak signal to noise ratio (PSNR) and feature similarity index (FSIM) are two quantitative performance metrics, which are utilized in this study [99]. PSNR measures the accuracy of the reconstructed image and is formulated as follows:

(25) $$\begin{array}{c} \text{PSNR}=10\log_{10}\left(\frac{255^{2}}{\left(1/mn\right)\sum_{j=1}^{m}\sum_{k=1}^{n}{\left(X_{j,k}-X_{j,k}^{'}\right)}^{2}}\right), \end{array}$$

where *mn* is the size of image and *X* and *X*′ are the main and the processed images, respectively. In addition, FSIM is a criterion that calculates the similarity of the thresholded and original images as follows:

(26) $$\begin{array}{c} \text{FISM}=\frac{\sum_{x\in X}S_{L}\left(x\right)PC_{m}\left(x\right)}{\sum_{x\in X}PC_{m}\left(x\right)}, \end{array}$$

where *S*_*L*_(*x*) shows the similarity of images and PC_*m*_(*x*) is the phase congruency map. Tables 31 and 32 present the values of PSNR and FSIM metrics, respectively. According to these results, the SNS algorithm achieved better PSNR and FSIM values for all thresholding levels.

This comprehensive study demonstrates that the developed SNS has competence among the other metaheuristic algorithms. Based on the results in solving classical engineering problems, it can be concluded that the SNS algorithm can perform superior to other algorithms in dealing with semi-real constrained problems. In addition, the image segmentation problem results show the SNS algorithm's ability to solve real-world problems.

## 4. Conclusion

The social network search (SNS) is a newly developed metaheuristic algorithm that mimics the behavior of social network users in expressing their opinions. In the present study, the SNS algorithm was employed for solving 14 semi-real constrained optimization problems and one real-world application in the field of remote sensing at a relatively low computational cost. From the comparative study, the SNS has shown its potential to handle various constrained optimization problems, and its performance is much better than other state-of-the-art algorithms in terms of the selected performance metrics. This is partly because there are no parameters to be fine-tuned in the SNS. In addition, it is worth mentioning that a simple penalty function method is used for constraint handling, while other compared methods maybe used advanced methods for this task.

This algorithm uses four moods of users in the social networks, namely, imitation, conversation, disputation, and innovation. Users are influenced to express their new views using these four moods simulated from real-world behaviors of users in social networks that randomly accrue for each of them. As further studies, different modifications can be employed to improve the performance of the SNS. Some of these editions are listed below:

- In the course of iterations, each user is affected during a randomly selected mood. Developing this random selection to an adaptive selection may affect the performance.
- In the imitation mood, the new view is created inside the imitation space. A new model for this space can have a high impact.
- The shock radius (*R*) and popularity radius (*r*) are two important key parameters for improving the imitation mood output.
- In the imitation, conversation, and innovation moods, a random user (*X*_*j*_) is selected. The selection of this user affects extremely the performance of the SNS. Another selection mechanism can be useful.
- The subject (*X*_*k*_) in conversation mood has an effective impact on the quality of the newly generated solutions.
- In conversation mood, the direction and size of movements are affected by sign(*f*_*i*_ − *f*_*j*_). The change of this factor in an adaptive way that affects the size of movements is desirable.
- In the disputation mood, a random number of users are considered. Different strategies can be integrated with this mood. For example, different neighborhood topologies can be used. In addition, dynamic regrouping schema can be useful to improve the performance of disputation mood.
- A new mood can be designed to improve the ability of the SNS by modeling another specific situation in social networks.

Hybridization of the proposed algorithm with other popular algorithms is a common way to benefit from the idea of different metaheuristics to develop a more robust optimization algorithm. In addition, the ability of this algorithm should be examined in dealing with other complex real-world optimization problems in different branches of science.

## Figures and Tables

**Table 1:** List of some popular and new metaheuristic algorithms.

Algorithm	References
Evolution strategy (ES)	[18]
Genetic algorithms (GA)	[19]
Ant colony optimization (ACO)	[20]
Particle swarm optimization (PSO)	[21]
Differential evolution (DE)	[22]
Cuckoo search (CS)	[23]
Bat algorithm (BA)	[24]
Charged system search (CSS)	[25]
Firefly algorithm (FA)	[26]
Eagle strategy (ES)	[27]
Krill herd algorithm (KH)	[28]
Flower pollination algorithm (FPA)	[29]
Grey wolf optimizer (GWO)	[30]
Optimization based on phylogram analysis (OPA)	[31]
Whale optimization algorithm (WOA)	[32]
Developed swarm optimizer (DSO)	[33]
Stochastic paint optimizer (SPO)	[34]
Chaos game optimization (CGO)	[35, 36]
Atomic orbital search (AOS)	[37, 38]
Material generation algorithm (MGA)	[39]
Crystal structure algorithm (CryStAl)	[40]
Social network search (SNS)	[41]

**Table 2:** Best results of the cantilever beam design example.

Algorithm	Variables					Constraint	
	*x*_1_	*x*_2_	*x*_3_	*x*_4_	*x*_5_	*g*_1_(*X*)	*f*(*X*)
CS [18]	6.0089	5.3049	4.5023	3.5077	2.1504	−6.448*E* − 05	1.33999
MFO [56]	5.98487	5.31672	4.49733	3.51361	2.16162	4.182*E* − 09	1.33998
ALO [57]	6.01812	5.31142	4.48836	3.49751	2.15832	−2.995*E* − 06	1.33995
SOS [58]	6.01878	5.30344	4.49587	3.49896	2.15564	1.393*E* − 04	1.33996
SSA [59]	6.01513	5.30930	4.49500	3.50142	2.15278	4.0578*E* − 09	1.33995
SNS (present study)	6.01545	5.31066	4.48800	3.50528	2.15428	−3.2718*E* − 07	1.33995

MFO: moth-flame optimization algorithm. ALO: ant lion optimizer. SSA: salp swarm algorithm.

**Table 3:** Comparative results of SNS with other methods for the cantilever beam design problem.

Algorithm	Worst	Mean	Best	SD	NFEs
SOS [58]	NA	1.33997	1.33996	1.1*E* − 5	15,000
CGO [37]	1.340602	1.340052	1.339970	1.2245*E* − 04	100,000
AOS [39]	1.491711	1.351954	1.339957	0.02499743	100,000
MGA [40]	1.3402011	1.3400526	1.3399756	6.99*E* − 05	100,000
SNS (present study)	1.3399576	1.3399576	1.3399576	1.1102*E* − 15	12,000

**Table 4:** Best results for the optimal design of I-shaped beam problem.

Algorithm	Variables				Constraints		
	*x*_1_	*x*_2_	*x*_3_	*x*_4_	*g*_1_(*X*)	*g*_2_(*X*)	*f*(*X*)
IARSM [60]	79.99	48.42	0.9	2.4	0.0869999	−1.524540	0.0131
CS [18]	80	50	0.9	2.3216	−0.012005	−1.570020	0.01307
GWO [61]	80	50	0.9	2.3217	−0.009059	−1.570071	0.0131
EMGO-FCR [61]	80	50	0.9	2.3200	−0.176000	−1.567179	0.0131
SOS [58]	80	50	0.9	2.3217	−0.000222	−1.570224	0.01307
AEFA-C [61]	79.9671	49.99	0.9	2.3164	−0.560371	−1.559518	0.0131
SNS (present study)	80	50	0.9	2.3217	0	−1.570228	0.0130741

IARSM: improved adaptive response surface method. EMGO-FCR: ensemble meta model-based global optimization using fuzzy clustering. AEFA-C: artificial electric field algorithm.

**Table 5:** Comparison of the results of SNS with the other methods for the I-shaped beam problem.

Algorithm	Worst	Mean	Best	SD	NFEs
CS [18]	0.01353646	0.0132165	0.0130747	0.0001345	5000
SOS [58]	NA	0.0130884	0.0130741	4.0*E* − 5	5000
AOS [39]	0.0138140	0.0131788	0.0130741	1.555*E* − 04	100,000
SNS (present study)	0.0130764	0.0130743	0.0130741	4.313*E* − 07	3600

**Table 6:** Best results of the three-bar truss design problem.

Algorithm	Variables		Constraints			
	*x*_1_	*x*_2_	*g*_1_(*X*)	*g*_2_(*X*)	*g*_3_(*X*)	*f*(*X*)
GA [62]	0.788915	0.407569	9.6430*E* − 07	−1.464873605	−0.53512542	263.8958857
PSO [62]	0.788669	0.408265	4.8650*E* − 07	−1.464082376	−0.535917137	263.8958434
ICA [62]	0.788625	0.408389	8.4180*E* − 07	−1.463941244	−0.536057913	263.8958452
CS [18]	0.78867	0.40902	−2.9000*E* − 04	−0.26853	−0.73176	263.9716
WCA [5]	0.788651	0.408316	0.0000*E* + 00	−1.464024	−0.535975	263.895843
GWO [62]	0.788648	0.408325	3.3400*E* − 08	−1.464014397	−0.535985569	263.8960063
ALO [57]	0.78866281	0.4082831	−5.3170*E* − 12	−1.464062005	−0.53593799	263.8958434
MFO [56]	0.78824477	0.4094669	7.7090*E* − 12	−1.462717072	−0.537282927	263.8959796
SSA [59]	0.78866541	0.4082757	3.0000*E* − 10	−1.464070360	−0.53592963	263.8958434
WSA [62]	0.788683	0.408227	−7.2400*E* − 07	−1.464126180	−0.53587454	263.8958434
SNS (present study)	0.78868473	0.4082211	3.2978*E* − 08	−1.4641325	−0.5358675	263.8958434

WCA: water cycle algorithm. WSA: water strider algorithm.

**Table 7:** Comparative results of SNS with other methods for the three-bar truss design problem.

Algorithm	Worst	Mean	Best	SD	NFEs
GA [62]	264.82080546	263.96803663	263.89588573	1.66862*E* − 01	50,000
PSO [62]	264.58490296	263.95741428	263.89584341	1.36897*E* − 01	50,000
ICA [62]	263.91413326	263.89932689	263.89584519	4.11693*E* − 03	50,000
CS [18]	NA	264.0669	263.97156	9.00000*E* − 05	15,000
WCA [5]	263.896201	263.895903	263.895843	8.71000*E* − 05	5250
GWO [62]	263.90421778	263.89795501	263.89600631	1.61422*E* − 03	50,000
WSA [62]	263.89743217	263.89606687	263.8958434	3.11960*E* − 04	50,000
CGO [37]	263.8960068	263.8958511	263.895843	2.51*E* − 05	100,000
AOS [39]	263.895845	263.895843	263.895843	8.26*E* − 09	100,000
SNS (present study)	263.8958561	263.8958462	263.8958434	3.31056*E* − 06	4800

**Table 8:** Best results of the tubular column example.

Algorithm	Variables		Constraints						
	*x*_1_	*x*_2_	*g*_1_(*X*)	*g*_2_(*X*)	*g*_3_(*X*)	*g*_4_(*X*)	*g*_5_(*X*)	*g*_6_(*X*)	*f*(*X*)
CS [18]	5.45139	0.29196	−0.0241	−0.1095	−0.633	−0.610	−0.315	−0.635	26.53217
ISA [63]	5.45115623	0.29196547	−2.5*E* − 10	−1.8*E* − 10	−0.6331	−0.6106	−0.3149	−0.635	26.5313
SNS	5.45115623	0.29196547	−2.6*E* − 10	−1.8*E* − 10	−0.6331	−0.6106	−0.3149	−0.635	26.4994969

**Table 9:** Comparative results of SNS with other methods for the tubular column example.

Algorithm	Worst	Mean	Best	SD	NFEs
ISA [63]	26.532	26.531	26.531	1.70*E* − 04	3000
CS [18]	26.53972	26.53504	26.53217	1.93*E* − 03	15,000
FA [64]	NA	28.74	26.52	2.08	3000
AOS [39]	26.60831361	26.53161399	26.53137828	1.0300*E* − 03	100,000
SNS (present study)	26.48637095	26.48636249	26.48636147	2.2160*E* − 06	1250

**Table 10:** Best results of the speed reducer design example.

	CS [18]	WCA [5]	BA [66]	ABC [67]	APSO [68]	SHO [69]	SNS
*x*_1_	3.50150	3.50000	3.50000	3.50000	3.50131	3.50159	3.50000
*x*_2_	0.70000	0.70000	0.70000	0.70000	0.70000	0.70000	0.70000
*x*_3_	17.00000	17.00000	17.00000	17.00000	18.00000	17.00000	17.00000
*x*_4_	7.60500	7.30000	7.30001	7.30000	8.12781	7.30000	7.30000
*x*_5_	7.81810	7.71532	7.71532	7.80000	8.04212	7.80000	7.71532
*x*_6_	3.35200	3.35021	3.35021	3.35022	3.35245	3.35127	3.35021
*x*_7_	5.28750	5.28665	5.28665	5.28668	5.28708	5.28874	5.28665
*g*_1_(*X*)	−0.07430	−0.07392	−0.07400	−0.07392	−0.12569	−0.07434	−0.07392
*g*_2_(*X*)	−0.19830	−0.19800	−0.19800	−0.19800	−0.28490	−0.19836	−0.19800
*g*_3_(*X*)	−0.43490	−0.49917	−0.49900	−0.49917	−0.34888	−0.49980	−0.49917
*g*_4_(*X*)	−0.90080	−0.90464	−0.90500	−0.90156	−0.89804	−0.90162	−0.90464
*g*_5_(*X*)	−0.00110	−0.66685	0.00000	0.00000	−0.66559	−0.66717	0.00000
*g*_6_(*X*)	−0.00040	0.00000	0.00000	−0.00063	−0.00026	−0.00117	0.00000
*g*_7_(*X*)	−0.70250	−0.70250	−0.70300	−0.70250	−0.68500	−0.70250	−0.70250
*g*_8_(*X*)	−0.00040	0.00000	0.00000	0.00000	−0.00038	−0.00045	0.00000
*g*_9_(*X*)	−0.58320	−0.58333	−0.58300	−0.58333	−0.58318	−0.58314	−0.58333
*g*_10_(*X*)	−0.08900	−0.05133	−0.05100	−0.05133	−0.14754	−0.05111	−0.05133
*g*_11_(*X*)	−0.01300	0.00000	0.00000	−0.01070	−0.04058	−0.01056	0.00000
*f*(*X*)	3000.98100	2994.47107	2994.46710	2997.05841	3187.63049	2998.55070	2994.47107

APSO: accelerated particle swarm optimization. SHO: spotted hyena optimizer.

**Table 11:** Comparative results of SNS with other methods for the speed reducer design example.

Algorithm	Worst	Mean	Best	SD	NFEs
CS [18]	3009.00	3007.1997	3000.981	4.96*E* + 00	250,000
ABC [67]	NA	2997.058412	2997.058412	0.00*E* + 00	30,000
WCA [5]	2994.505578	2994.474392	2994.471066	7.40*E* − 03	15,150
APSO [68]	4443.01763900	3822.64062400	3187.63048600	3.66*E* + 02	30,000
SHO [69]	3003.889	2999.64	2998.5507	1.93*E* + 00	NA
SSA	3015.662612	3005.574377	2996.021720	4.63*E* + 00	NA
WOA	3233.598124	3042.915023	2996.604340	4.08*E* + 01	NA
CSS	3106.216451	3005.658912	2996.492478	4.86*E* + 00	NA
CGO [37]	2995.504933	2994.465397	2994.443649	0.110282	100,000
FACSS [70]	3006.419746	2999.413798	2996.3752376	4.82*E* + 00	NA
SNS (present study)	2994.4710992	2994.4710696	2994.4710662	7.00*E* − 06	3750

FACSS: fuzzy adaptive charged system search.

**Table 12:** Best results of the piston lever example.

	PSO [71]	DE [71]	GA [71]	HPSO [71]	CS [18]	SNS
*x*_1_	133.3	129.4	250.0	135.5	0.050	0.050
*x*_2_	2.44	2.43	3.96	2.48	2.043	2.042
*x*_3_	117.14	119.80	60.03	116.62	120.000	120.000
*x*_4_	4.75	4.75	5.91	4.75	4.085	4.083
*g*_1_(*X*)	NA	NA	NA	NA	−1744.912	0.000
*g*_2_(*X*)	NA	NA	NA	NA	−600,000	−600,000
*g*_3_(*X*)	NA	NA	NA	NA	−117.185	−117.187
*g*_4_(*X*)	NA	NA	NA	NA	−4.50*E* − 04	−9.74*E* − 12
*f*(*X*)	122	159	161	162	8.427	8.412698349

**Table 13:** Comparative results of SNS with other methods for the piston lever example.

Algorithm	Worst	Mean	Best	SD	NFEs
PSO [71]	294	166	122	51.7	50,000
DE [71]	199	187	159	14.2	50,000
GA [71]	216	185	161	18.2	50,000
HPSO [71]	197	187	162	13.4	50,000
HPSO with Q-learning [71]	168	151	129	13.4	50,000
CS [18]	168.5920	40.2319	8.4271	59.0552	50,000
ISA [63]	610.6	226.5	8.4	111.2	12,500
CGO [37]	167.4728087	45.0486599	8.412813813	67.24763	100,000
AOS [39]	167.6649862	33.7412759	8.419142742	93.46674724	100,000
MGA [40]	167.4732134	32.4688925	8.413406652	29.96370439	100,000
SNS (present study)	167.4727747	24.3189743	8.412698349	47.71792646	5000

**Table 14:** Best results of the corrugated bulkhead design.

	CS [18]	VIGMM3 [61]	AEFA-C [61]	SNS
*x*_1_	37.1179498	57.69231	57.69277	57.69230732
*x*_2_	33.0350210	34.14762	34.13296	34.14762029
*x*_3_	37.1939476	57.69231	57.55294	57.69230729
*x*_4_	0.7306255	1.05000	1.05007	1.05
*g*_1_(*X*)	−23.3537699	−0.25839	−240.89634	−240.6946226
*g*_2_(*X*)	−15.9738532	−2.220*E* − 16	−11.59051	−1.46827*E* − 05
*g*_3_(*X*)	−0.00158548	−9.769*E* − 15	−6.864*E* − 05	−5.80038*E* − 09
*g*_4_(*X*)	−0.00039992	−5.551*E* − 16	−2.250*E* − 03	−6.20877*E* − 09
*g*_5_(*X*)	0.3193745	0.00000	−7.581*E* − 05	−6.50591*E* − 13
*g*_6_(*X*)	−4.1589266	0.68949	−23.41997	−23.544687
*f*(*X*)	5.894331	6.84296	−6.84584	6.842958019

VIGMM3: vibration-based ideal gas molecular movement. AEFA-C: artificial electric field algorithm.

**Table 15:** Comparative results of SNS with other methods for the corrugated bulkhead design.

Algorithm	Worst	Mean	Best	SD	NFEs
FA [64]	NA	10.23	7.21	1.95	12,000
LF-FA [64]	NA	8.83	6.95	1.26	12,000
LS-LF-FA [64]	NA	7.44	6.86	0.67	12,000
AD-IFA [64]	NA	7.21	6.84	0.58	12,000
AOS [39]	7.066936186	7.060808377	6.84295801	6.4911*E* − 04	100,000
SNS (present study)	6.843074399	6.842979802	6.842960515	2.0942*E* − 05	3125

LF-FA: Levy flight firefly algorithm. LS-LF-FA: logarithmic spiral path flight firefly. AD-IFA: spiral-Levy flight firefly algorithm.

**Table 16:** Best results of the pressure vessel design.

	G-QPSO [73]	HPSO [74]	CPSO [75]	CDE [76]	SAP [77]	ABC [67]	CS [18]	EO [78]
*x*_1_	0.81250	0.81250	0.81250	0.81250	0.81250	0.81250	0.81250	0.81250
*x*_2_	0.43750	0.43750	0.43750	0.43750	0.43750	0.43750	0.43750	0.43750
*x*_3_	42.09840	42.09840	42.09130	42.09840	40.32390	42.09845	42.09845	42.09845
*x*_4_	176.63720	176.63660	176.74650	176.63760	200.00000	176.63660	176.63660	176.63660
*g*_1_(*X*)	0.00000	0.00000	0.00000	0.00000	−0.03432	0.00000	0.00000	0.00000
*g*_2_(*X*)	−0.03580	−0.03580	−0.00036	−0.03580	−0.05285	−0.03588	−0.03588	−0.03588
*g*_3_(*X*)	−0.21790	3.12260	−118.76870	−3.70512	−27.10585	−0.00023	−0.00005	−0.00005
*g*_4_(*X*)	−63.36280	−63.36340	−63.25350	−63.36230	−40.00000	−63.36340	−63.36340	−63.36340
*f*(*X*)	6059.7208	6059.7143	6061.0777	6059.7340	6288.7445	6059.7143	6059.7143	6059.71430

	MFO [56]	GWO [31]	WOA [33]	APSO [68]	IAPSO [68]	NDE [79]	MCEO [80]	SNS
*x*_1_	0.8125	0.812500	0.812500	0.8125	0.8125	0.8125	0.8125	0.81250
*x*_2_	0.4375	0.434500	0.437500	0.4375	0.4375	0.4375	0.4375	0.43750
*x*_3_	42.098445	42.089181	42.0982699	42.0984	42.0984	42.0984455	42.0984455	42.09845
*x*_4_	176.636596	176.758731	176.638998	176.6374	176.6366	176.636595	176.636596	176.63660
*g*_1_(*X*)	−1.15*E* − 08	−0.00017880	−3.39*E* − 06	−8.80*E* − 07	−4.09*E* − 13	−1.4*E* − 15	−1.13*E* − 10	0.00000
*g*_2_(*X*)	−0.03588083	−0.03296921	−0.03588250	−0.03588126	−3.58*E* − 2	−0.00035880	−0.037564	−0.03588
*g*_3_(*X*)	0.04023295	−40.6168247	−1.25270175	−1.33153860	−1.39*E* − 07	−0.00000001	−4.73*E* − 04	0.00000
*g*_4_(*X*)	−63.363404	−63.241269	−63.361002	−63.3626	−63.3634	−0.63363404	−63.3634	−63.36340
*f*(*X*)	6059.7143	6051.5639	6059.7410	6059.72418	6059.71433	6059.71433	6059.7143	6059.71434

G-QPSO: Gaussian quantum-behaved particle swarm optimization. CPSO: co-evolutionary particle swarm optimization. CDE: co-evolutionary differential evolution. SAP: self-adaptive penalty approach. EO: equilibrium optimizer. MCEO: multilevel cross entropy optimizer.

**Table 17:** Comparative results of SNS with other methods for the pressure vessel design.

Algorithm	Worst	Mean	Best	SD	NFEs
SAP [77]	6308.15	6293.843	6288.745	7.41*E* + 00	3000
HPSO [74]	6288.677	6099.9323	6059.7143	8.62*E* + 01	81,000
CDE [76]	6059.734	6085.23	6371.046	4.30*E* + 01	204,800
CPSO [75]	6363.8041	6147.1332	6061.0777	8.65*E* + 01	200,000
PSO [73]	14076.324	8756.6803	6693.7212	1.49*E* + 03	8000
QPSO [73]	8017.2816	6839.9326	6059.7209	4.79*E* + 02	8000
G-QPSO [73]	7544.4925	6440.3786	6059.7208	4.48*E* + 02	8000
ABC [67]	NA	6245.308144	6059.714736	2.05*E* + 02	30,000
CS [18]	6495.347	6447.736	6059.714335	5.03*E* + 02	15,000
WOA [33]	NA	6068.05	6059.741	6.57*E* + 01	6300
APSO [68]	7544.49272	6470.71568	6059.7242	3.27*E* + 02	200,000
EO [78]	7544.4925	6668.114	6059.7143	5.66*E* + 02	15,000
CGO [37]	6330.958685	6250.957354	6247.672819	1.07*E* + 01	100,000
SNS (present study)	6410.086886	6097.100294	6059.714335	9.28*E* + 01	6000

**Table 18:** Best results of tension/compression spring design.

Algorithm	Variables			Constraints				
	*x*_1_	*x*_2_	*x*_3_	*g*_1_(*X*)	*g*_2_(*X*)	*g*_3_(*X*)	*g*_4_(*X*)	*f*(*X*)
CPSO [75]	0.051728	0.357644	11.244543	−8.25*E* − 04	−2.52*E* − 05	−4.051306	−0.727085	0.012674
WCA [5]	0.051680	0.356500	11.300400	−1.60*E* − 13	−7.90*E* − 14	−4.053300	−0.727800	0.012665
ABC [67]	0.051749	0.358179	11.203763	0.00*E* + 00	0.00*E* + 00	−4.056663	−0.726713	0.012665
SFOA [65]	0.051800	0.359000	11.279000	−3.24*E* − 06	−3.58*E* − 07	−4.060000	−0.726000	0.012700
APSO [68]	0.052588	0.378343	10.138862	−1.55*E* − 04	−8.33*E* − 04	−4.089171	−1.069069	0.012700
IAPSO [68]	0.051685	0.356629	11.294175	−1.97*E* − 10	−4.64*E* − 10	−4.053610	−1.091686	0.012665
MFO [56]	0.051994	0.364109	10.868421	−4.10*E* − 06	3.04*E* − 06	−4.068140	−0.722600	0.012667
GWO [31]	0.051690	0.356737	11.288850	−7.91*E* − 05	−7.51*E* − 06	−4.053380	−0.727720	0.012666
WOA [33]	0.051200	0.345200	12.004000	−5.60*E* − 04	−3.00*E* − 05	−4.027400	−0.735700	0.012676
SHO [69]	0.051144	0.343751	12.095500	−3.30*E* − 04	1.16*E* − 05	−4.025790	−0.736740	0.012674
NDE [79]	0.051689	0.356718	11.288968	0.00*E* + 00	0.00*E* + 00	−4.053785	−0.727728	0.012665
SSA [59]	0.051207	0.345215	12.004032	−5.60*E* − 04	−3.70*E* − 05	−4.027410	−0.735720	0.012676
SNS	0.051587	0.354268	11.434058	−1.37*E* − 08	−3.18*E* − 10	−4.048919	−0.729430	0.012665

**Table 19:** Comparative results of tension/compression spring design.

Algorithm	Worst	Mean	Best	SD	NFEs
CPSO [75]	0.012924	0.01273	0.0126747	5.20*E* − 05	200,000
HPSO [74]	0.0127191	0.0127072	0.0126652	1.58*E* − 05	81,000
CDE [76]	0.01279	0.012703	0.0126702	2.70*E* − 05	204,800
PSO [73]	0.071802	0.019555	0.012857	1.17*E* − 02	20,000
QPSO [73]	0.018127	0.013854	0.012669	1.34*E* − 03	20,000
G-QPSO [73]	0.015869	0.012996	0.012666	6.28*E* − 04	20,000
WCA [5]	0.012952	0.012746	0.012665	8.06*E* − 05	11,750
ABC [67]	NA	0.012709	0.012665	1.28*E* − 02	30,000
APSO [68]	0.014937	0.013297	0.0127	6.85*E* − 04	120,000
IAPSO [68]	0.01782864	0.01367653	0.01266523	1.57*E* − 03	20,000
WOA [33]	NA	0.0127	0.0126763	3.00*E* − 04	4410
MCEO [80]	0.01350901	0.0127196	0.01266051	3.79*E* − 05	2000
EO [78]	0.013997	0.013017	0.012666	3.91*E* − 04	15,000
SNS (present study)	0.012765873	0.012684717	0.012665246	2.38549*E* − 05	9000

**Table 20:** Best results of welded beam design.

	BBO [62]	PSO [62]	ICA [62]	WCA [5]	ABC [67]	EO [78]	TEO [82]	SSA [59]
*x*_1_	0.1854860	0.219292	0.205799	0.205728	0.205730	0.2057000	0.20568100	0.2057000
*x*_2_	4.3129000	3.430416	3.469634	3.470522	3.470489	3.4705000	3.47230500	3.4714000
*x*_3_	8.4399030	8.433559	9.034950	9.036620	9.036624	9.0366000	9.03513300	9.0366000
*x*_4_	0.2359020	0.236204	0.205806	0.205729	0.205730	0.2057000	0.20579600	0.2057000
*g*_1_(*X*)	−114.190503	−0.193642	0.020688	0.010832	0.000000	0.0094704	−0.60927688	−0.8256803
*g*_2_(*X*)	−6.7171616	0.017873	−0.017763	0.119259	−0.000002	0.0014958	0.22468860	−0.7106346
*g*_3_(*X*)	−0.0504000	−0.016900	−0.000007	−0.000001	0.000000	−0.0000001	−0.00011500	0.0000000
*g*_4_(*X*)	−3.2078633	−3.276396	−3.390414	−3.390662	−3.432984	−3.3906592	−3.39027259	−3.3908203
*g*_5_(*X*)	−0.0604860	−0.094292	−0.080799	−0.080728	−0.080730	−0.0807295	−0.08068100	−0.0807000
*g*_6_(*X*)	−0.2350000	−0.235000	−0.236000	−0.235540	−0.235540	−0.2355403	−0.23553783	−0.2355381
*g*_7_(*X*)	−2639.74280	−2668.48570	−5.951908	0.057684	0.000000	0.0008607	−5.15644919	−2.6033472
*f*(*X*)	1.9180550	1.852720	1.725135	1.724856	1.724852	1.7249000	1.72528400	1.7249100

	WSA [62]	MCEO [80]	CBO [62]	GWO [62]	SHO [69]	WOA [33]	CCSA [83]	SNS
*x*_1_	0.20573000	0.20572964	0.2057220	0.2056770	0.2055630	0.2053960	0.2057000	0.2057296
*x*_2_	3.47048900	3.47048866	3.4704100	3.4708940	3.4748460	3.4842930	3.4702000	3.4704887
*x*_3_	9.03662400	9.03662391	9.0372760	9.0385580	9.0357990	9.0374260	9.0362000	9.0366239
*x*_4_	0.20573000	0.20572964	0.2057350	0.2057390	0.2058110	0.2062760	0.2057000	0.2057296
*g*_1_(*X*)	−0.02539959	0.00000363	−0.0246881	−0.0519206	−1.4028657	−21.5450190	−0.0020610	0.0000000
*g*_2_(*X*)	−0.05312238	−0.00002880	−5.1106833	−14.2018357	−6.3837568	−84.7713395	−0.6569560	0.0000000
*g*_3_(*X*)	0.00000000	0.00000000	−0.0000130	−0.0000620	−0.0002480	−0.0008800	−0.0000045	0.0000000
*g*_4_(*X*)	−3.39065616	−3.39000000	−3.3905161	−3.3902412	−3.3898697	−3.3852837	−3.3906250	−3.3906591
*g*_5_(*X*)	−0.08073000	−0.08072964	−0.0807220	−0.0806770	−0.0805630	−0.0803960	−0.0807296	−0.0807296
*g*_6_(*X*)	−0.23554035	−0.23554032	−0.2360000	−0.2355503	−0.2355421	−0.2355825	−0.2355406	−0.2355403
*g*_7_(*X*)	−0.03155555	−0.00001860	−0.7536625	−1.6632990	−6.7607624	−48.2829292	−0.3906801	0.0000000
*f*(*X*)	1.72485200	1.72485230	1.7246630	1.7252320	1.7256610	1.7304990	1.7249000	1.7248523

CCSA: conscious neighborhood-based crow search algorithm.

**Table 21:** Comparative results of welded beam design.

Algorithm	Worst	Mean	Best	SD	NFEs
PSO [62]	3.841845	2.613785	1.85272	4.71*E* − 01	50,000
CPSO [75]	1.782143	1.748831	1.728024	1.29*E* − 02	200,000
HPSO [74]	1.814295	1.749040	1.724852	4.00*E* − 02	81,000
CDE [76]	1.824105	1.768158	1.733461	2.22*E* − 02	204,800
ICA [62]	2.237755	1.79433	1.725135	1.10*E* − 01	50,000
BBO [62]	3.606933	2.630412	1.918055	4.11*E* − 01	50,000
FA [54]	2.345579	1.878656	1.731206	2.68*E* − 01	50,000
CSS [26]	1.759479	1.739654	1.724866	8.06*E* − 03	NA
WCA [5]	1.744697	1.726427	1.724856	4.29*E* − 03	46,500
ABC [67]	NA	1.741913	1.724852	3.10*E* − 02	30,000
MFO [62]	1.724852	1.732109	1.950241	3.40*E* − 02	50,000
SCA [62]	1.786863	1.849364	1.925162	3.47*E* − 02	50,000
APSO [68]	1.993999	1.877851	1.736193	7.61*E* − 02	50,000
SHO [69]	1.726064	1.725828	1.725661	2.87*E* − 04	NA
SSA [62]	1.725886	1.823426	2.246638	1.28*E* − 01	50,000
GWO [62]	1.725232	1.72631	1.728487	7.71*E* − 04	50,000
WOA [33]	NA	1.7320	1.730499	0.0226	9900
TEO [82]	1.931161	1.768040	1.725284	5.82*E* − 02	NA
EO [78]	1.736725	1.726482	1.724853	3.26*E* − 03	15,000
WSA [62]	1.725068	1.724908	1.724852	4.15*E* − 05	50,000
FACSS [70]	1.736565	1.730223	1.724853	6.363*E* − 7	100,000
T-CSS [84]	1.735656	1.730212	1.724860	2.00*E* − 06	100,000
SNS (present study)	1.725051	1.724880	1.724852	5.18*E* − 05	9000

T-CSS: tribe-charged system search.

**Table 22:** Best results of the gear train design.

Algorithm	Variables				*f*(*X*)
	*x*_1_	*x*_2_	*x*_3_	*x*_4_	
GA [62]	49	19	16	43	2.70*E* − 12
PSO [62]	34	13	20	53	2.31*E* − 11
ICA [62]	43	16	19	49	2.70*E* − 12
CS [18]	43	16	19	49	2.70*E* − 12
ABC [67]	49	16	19	43	2.70*E* − 12
MSFWA [86]	49	19	16	43	2.70*E* − 12
MBA [87]	43	16	19	49	2.70*E* − 12
BBO [62]	53	26	15	51	2.31*E* − 11
NNA [62]	49	16	19	43	2.70*E* − 12
GWO [62]	49	19	16	43	2.70*E* − 12
ISA [63]	43	19	16	49	2.70*E* − 12
APSO [68]	43	16	19	49	2.70*E* − 12
IAPSO [68]	43	16	19	49	2.70*E* − 12
MVO [88]	43	16	19	49	2.70*E* − 12
MFO [56]	43	19	16	49	2.70*E* − 12
ALO [57]	49	19	16	43	2.70*E* − 12
PSOSCALF [89]	49	19	16	43	2.70*E* − 12
WSA [62]	43	16	19	49	2.70*E* − 12
SNS (present study)	43	19	16	49	2.70085714*E* − 12

MBA: mine blast algorithm.

**Table 23:** Comparative results of SNS with other methods for the gear train design.

Algorithm	Worst	Mean	Best	SD	NFEs
GA [62]	1.5247*E* − 08	1.6212*E* − 09	2.7009*E* − 12	3.2174*E* − 09	50,000
PSO [62]	1.0222*E* − 06	7.9383*E* − 08	2.3078*E* − 11	1.8147*E* − 07	50,000
ICA [62]	2.3576*E* − 09	8.0417*E* − 10	2.7009*E* − 12	7.7862*E* − 10	50,000
UPSO [90]	N.A	3.80562*E* − 08	2.700857*E* − 12	1.09*E* − 07	100,000
MFO [62]	2.7265*E* − 08	7.5337*E* − 09	2.3078*E* − 11	9.3539*E* − 09	50,000
SCA [62]	2.3576*E* − 09	8.8113*E* − 10	2.7009*E* − 12	6.4529*E* − 10	50,000
SSA [62]	2.7265*E* − 08	1.9822*E* − 09	2.7009*E* − 12	4.5748*E* − 09	50,000
BBO [62]	4.2018*E* − 07	4.5418*E* − 08	2.3078*E* − 11	7.2953*E* − 08	50,000
CBO [62]	1.1173*E* − 08	2.1032*E* − 09	2.3078*E* − 11	2.4025*E* − 09	50,000
NNA [62]	2.3576*E* − 09	6.1128*E* − 10	2.7009*E* − 12	6.1167*E* − 10	50,000
GWO [62]	9.9216*E* − 10	3.3777*E* − 10	2.7009*E* − 12	4.0956*E* − 10	50,000
WOA [62]	6.5123*E* − 09	9.6633*E* − 10	2.7009*E* − 12	1.1296*E* − 09	50,000
WSA [62]	1.3616*E* − 09	1.6800*E* − 10	2.7009*E* − 12	3.8265*E* − 10	50,000
SNS (present study)	1.36165*E* − 09	1.68012*E* − 10	2.700857*E* − 12	3.74894*E* − 10	25,000

**Table 24:** Best results of a reinforced concrete beam design.

	GHN-EP [71]	GA [92]	FA [54]	CS [18]	ISA [63]	AOS [39]	SNS
*x*_1_	6.32	7.20	6.32	6.32	6.32	6.32	6.32
*x*_2_	34	32	34	34	34	34	34
*x*_3_	8.637180	8.0451	8.5000	8.5000	8.5000	8.5	8.5
*g*_1_(*X*)	−0.7745	−2.8779	−0.2241	−0.2241	−0.2241	−0.22409498	−0.22409411
*g*_2_(*X*)	−0.0635	−0.0224	0	0	0	−1.00*E* − 07	5.27667*E* − 12
*f*(*X*)	362.00648	366.1459	359.2080	359.2080	359.2080	359.2080	359.2080

GHN-EP: generalized Hopfield network-based extended penalty approach.

**Table 25:** Comparative results of SNS with other methods for a reinforced concrete beam design.

Algorithm	Worst	Mean	Best	SD	NFEs
FA [54]	669.150	460.706	359.2080	80.73870	25,000
CS [18]	NA	NA	359.2080	NA	5000
AOS [39]	362.2535	359.3306872	359.2080	0.59614901	100,000
SNS (present study)	362.634	359.3222001	359.2080	0.61498581	1000

**Table 26:** Best results of the car side impact design example.

	PSO [54]	DE [54]	GA [54]	CS [18]	BA [66]	SNS
*x*_1_	0.50000	0.50000	0.50005	0.50000	0.50000	0.5
*x*_2_	1.11670	1.11670	1.28017	1.11643	1.11670	1.115933208
*x*_3_	0.50000	0.50000	0.50001	0.50000	0.50000	0.5
*x*_4_	1.30208	1.30208	1.03302	1.30208	1.30208	1.302918991
*x*_5_	0.50000	0.50000	0.50001	0.50000	0.50000	0.5
*x*_6_	1.50000	1.50000	0.50000	1.50000	1.50000	1.5
*x*_7_	0.50000	0.50000	0.50000	0.50000	0.50000	0.5
*x*_8_	0.34500	0.34500	0.34994	0.34500	0.34500	0.345
*x*_9_	0.19200	0.19200	0.19200	0.19200	0.19200	0.192
*x*_10_	−19.54935	−19.54935	10.3119	−19.54935	−19.54935	−19.6388662
*x*_11_	−0.00431	−0.00431	0.00167	−0.00431	−0.00431	1.49192*E* − 06
*g*_1_(*X*)	−0.617505	−0.617505	−0.43167	−0.617423	−0.61750525	−0.61849499
*g*_2_(*X*)	−0.092707	−0.092707	−0.09839	−0.092703	−0.09270723	−0.09273231
*g*_3_(*X*)	−0.100627	−0.100627	−0.13685	−0.100625	−0.10062705	−0.10061401
*g*_4_(*X*)	−0.034209	−0.034209	−0.02699	−0.034182	−0.03420961	−0.03418562
*g*_5_(*X*)	−4.278328	−4.278328	−3.77008	−4.277761	−4.27832783	−4.28314394
*g*_6_(*X*)	−7.2838105	−7.2838105	−3.36103	−7.282103	−7.28381045	−7.29404065
*g*_7_(*X*)	−0.0026365	−0.0026365	−0.00025	3.647*E* − 05	−0.00263652	0
*g*_8_(*X*)	−2.425*E* − 05	−2.425*E* − 05	−8.6156*E* − 06	1.395*E* − 06	−2.43*E* − 05	0
*g*_9_(*X*)	−0.9654269	−0.9654269	−0.58567	−0.965154	−0.96542696	−0.96669762
*g*_10_(*X*)	−0.1666041	−0.1666041	0.502506	−0.166604	−0.16660413	−0.16739262
*f*(*X*)	22.84474	22.84298	22.85653	22.84294	22.84474	22.84297965

**Table 27:** Comparative results of SNS with other methods for the car side impact design example.

Algorithm	Worst	Mean	Best	SD	NFEs
PSO [54]	23.21354	22.89429	22.84474	0.15017	20,000
DE [54]	24.12606	23.22828	22.84298	0.34451	20,000
GA [54]	26.240578	23.51585	22.85653	0.66555	20,000
BA [66]	23.21354	22.89273	22.84474	0.17383	20,000
FA [54]	24.06623	22.89376	22.84298	0.16667	20,000
CS [18]	23.25998	22.85858	22.84294	0.07612	20,000
WCA [94]	23.37093376	22.97516442	22.84303648	1.9772*E* − 01	NA
MBA [94]	23.48894217	22.93642104	22.84359640	1.5258*E* − 01	NA
ER-WCA [94]	24.45531280	23.06992534	22.84326461	3.502*E* − 01	NA
WOA [94]	27.36081368	24.81448617	23.04216220	9.657*E* − 01	NA
FACSS [70]	23.05362562	22.91212354	22.84907401	4.726*E* − 02	NA
CSS [84]	24.863563	23.523265	23.007336	0.562345	100,000
T-CSS [84]	23.800904	22.903653	22.847848	0.078565	100,000
SNS (present study)	23.18454939	22.88145736	22.84296954	0.101801211	20,000

ER-WCA: evaporation rate-based water cycle algorithm.

**Table 28:** Best results of the cantilever stepped beam.

	FA [54]	TEO [82]	PSO [82]	SNS (present study)
*x*_1_	3	3	5	3
*x*_2_	3.1	3.1	3.1	3.1
*x*_3_	2.6	2.6	2.6	2.6
*x*_4_	2.205	2.21629046531169	2.204556	2.204555692
*x*_5_	1.750	1.76910085340763	1.749757	1.749757012
*x*_6_	60	60	47	60
*x*_7_	55	55	55	55
*x*_8_	50	50	50	50
*x*_9_	44.091	44.014420969625	44.09111	44.09111383
*x*_10_	34.995	34.8445777966116	34.995140	34.99514024
*g*_1_(*X*)	−1.83171281	−33.157060	−1.49*E* − 06	−1.81899*E* − 12
*g*_2_(*X*)	−2.74873225	−25.554426	−2.38*E* − 06	−1.81899*E* − 12
*g*_3_(*X*)	−153.846153	−153.846154	−153.8461538	−153.8461538
*g*_4_(*X*)	−1203.41242	−1203.412423	−1203.412423	−1203.412423
*g*_5_(*X*)	−111.111111	−111.111111	−419.1942055	−111.1111111
*g*_6_(*X*)	−2.44816450	−2.447883	−2.44430588	−2.448137336
*g*_7_(*X*)	−0.00285714	−0.303792	0	−3.19744*E* − 14
*g*_8_(*X*)	−0.00408163	−0.140500	−4.54*E* − 09	−3.55271*E* − 15
*g*_9_(*X*)	−0.76923076	−0.769231	−0.769230769	−0.769230769
*g*_10_(*X*)	−2.25806451	−2.258065	−2.258064516	−2.258064516
*g*_11_(*X*)	0	0	−10.6	0
*f*(*X*)	63893.52	63994.018919	69393.430796	63893.4307958715

**Table 29:** Comparative results of SNS with other methods for the cantilever stepped beam.

Algorithm	Worst	Mean	Best	SD	NFEs
FA [54]	64262.99420	64144.75312	63893.52578	175.91879	50,000
PSO [82]	NA	NA	69393.430796	NA	300,000
TEO [82]	NA	NA	63994.018919	NA	300,000
SNS (present study)	63905.69523997	63893.88749760	63893.43079588	2.21*E* + 00	20,000

**Table 30:** Comparison of threshold values between algorithms.

*n*	Methods		
	PSO [96]	DPSO [96]	ABC [96]
5	40 82 123 164 207	43 86 128 171 211	40 82 121 162 205
	62 100 138 176 217	65 104 142 180 217	63 100 139 177 217
	54 93 131 171 210	51 95 99 147 166	56 95 134 173 214

7	30 60 90 119 151 184 220	33 66 97 129 160 191 221	33 65 95 126 157 188 220
	52 80 105 133 161 189 219	54 82 109 137 166 195 225	54 81 109 137 166 195 225
	44 74 104 132 161 190 219	43 73 103 132 161 190 219	46 76 104 132 161 190 217

9	22 44 68 94 120 148 175 203 231	22 45 70 96 121 146 171 197 224	22 48 73 99 125 152 179 204 231
	47 68 89 110 132 155 180 203 229	44 63 82 103 125 147 171 197 226	29 51 71 94 121 147 171 197 228
	21 48 75 101 128 155 181 206 230	21 47 72 95 119 145 172 199 225	21 48 75 101 126 151 177 201 226

11	17 38 59 78 98 119 140 162 183 207 231	16 35 54 74 94 116 139 163 186 209 231	16 32 50 70 91 113 136 159 182 206 231
	28 46 66 84 101 118 135 153 175 198 224	44 63 82 101 119 138 157 176 194 214 234	28 46 66 86 106 126 144 164 184 204 225
	18 36 56 77 99 121 141 161 181 202 226	18 39 59 80 101 122 143 164 186 208 230	18 37 56 76 96 118 140 162 185 208 230

*n*	CS [96]	CS_McCulloch_ [96]	SNS (present study)
5	48 89 132 181 210	45 88 130 172 208	45 88 131 174 215
	49 91 133 179 215	47 89 132 178 212	43 57 71 97 173
	51 93 140 182 219	48 88 133 173 209	56 95 134 174 214

7	42 81 110 145 182 221 245	39 76 107 139 173 204 232	35 66 96 126 157 189 220
	42 83 115 143 179 208 239	40 77 108 142 174 203 233	40 50 51 66 116 182 206
	43 77 109 143 181 217 242	41 76 106 140 175 205 234	46 75 103 132 161 191 223

9	34 61 79 110 133 159 189 210 233	25 51 75 103 130 156 182 209 235	24 47 72 97 122 148 174 200 225
	25 56 77 109 137 161 191 210 240	20 53 70 108 135 159 189 211 238	12 12 14 48 62 115 157 186 191
	30 62 79 110 132 164 198 214 245	29 61 75 109 130 163 194 213 240	18 47 74 99 123 148 173 198 226

11	28 47 65 87 105 132 145 167 191 220 239	23 45 64 84 105 129 145 164 186 211 235	21 41 63 83 103 123 143 165 187 209 232
	25 48 69 91 110 132 147 165 191 214 240	24 46 67 89 107 131 146 162 188 213 238	11 12 40 61 83 100 110 180 217 219 251
	28 50 66 85 108 135 148 167 189 211 235	22 48 65 85 106 130 147 163 185 212 235	16 34 54 74 95 117 139 161 183 206 230

**Table 31:** Comparison of PSNR between algorithms.

*n*	PSO [96]	DPSO [96]	ABC [96]	CS [96]	CS_McCulloch_ [96]	SNS
5	20.3884	20.4267	20.4707	20.4781	20.4816	**26.0094**
7	21.1449	21.1886	21.2327	21.155	21.316	**26.1077**
9	22.2473	22.3048	22.3342	22.5418	22.7496	**26.7082**
11	23.139	23.2316	23.3399	23.5449	23.6527	**27.6536**

**Table 32:** Comparison of FSIM between algorithms.

*n*	PSO [96]	DPSO [96]	ABC [96]	CS [96]	CS_McCulloch_ [96]	SNS
5	0.9425	0.9427	0.9433	0.9402	0.9445	0.96655
7	0.9097	0.9125	0.9141	0.9071	0.9174	0.97626
9	0.947	0.9479	0.9486	0.9441	0.9549	0.98178
11	0.9175	0.9179	0.9214	0.9156	0.9276	0.98441

## Data Availability

The data used to support the findings of this study are included within the article.
